# Supplementary material for: DHX9 sustains hematopoietic stem cell function in cooperation with H3 acetylation
Source: Stem Cell Reports. 2026 Feb 5;21(3):102794. doi: 10.1016/j.stemcr.2026.102794 (PMC12985375; doi:10.1016/j.stemcr.2026.102794)
Supplement: Document S1. Figures S1–S6 and Tables S1–S3 [file mmc1.pdf]

Stem Cell Reports, Volume *21*

Supplemental Information

DHX9 sustains hematopoietic stem cell function in cooperation with H3 acetylation

Minhui Shi, Mengqing Gao, Huixin Luo, Chong Wang, Xueyang Hu, Yacen Xiong, Yan Chen, Xingxing Ren, Shu Zhu, and Huaiping Zhu

Supplemental Information

DHX9 sustains hematopoietic stem cell function in cooperation with H3 acetylation

Minhui Shi, Mengqing Gao, Huixin Luo, Chong Wang, Xueyang Hu, Yacen Xiong, Yan Chen, Xingxing Ren, Shu Zhu, and Huaiping Zhu

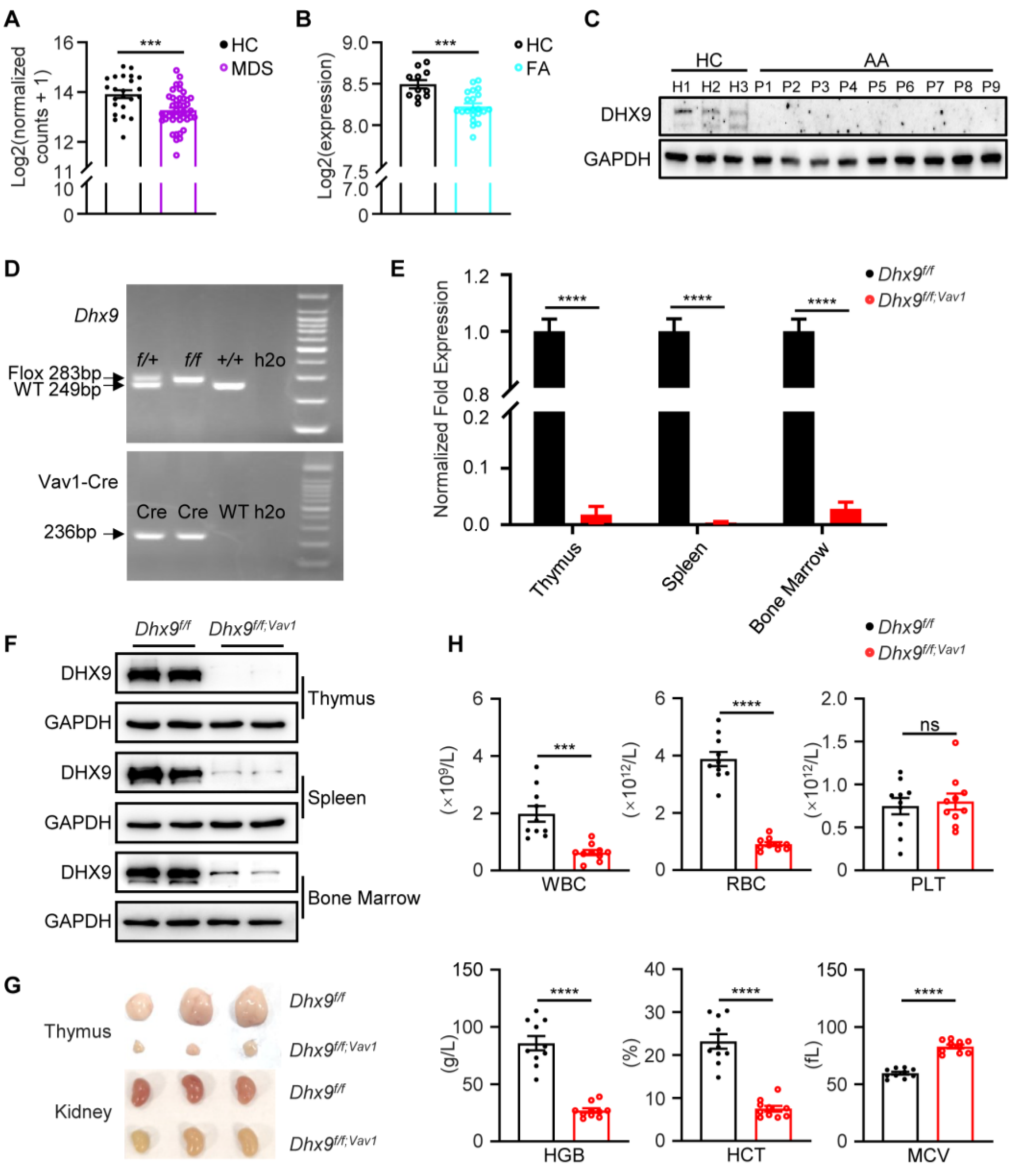

Figure S1. Generation of hematopoietic-specific *Dhx9* conditional knockout mice, related to Figure 1.

(A) RNA-seq data of CD34$^+$ cells from healthy controls (HC) (n = 23) and myelodysplastic syndromes (MDS) (n = 44) patients were obtained from the GEO database (GSE111085).

(B) mRNA expression profiles of BM cells from healthy controls and patients with fanconi anemia (FA) were obtained using the Affymetrix GPL96 microarray platform (GSE16334).

(C) Western blot showing DHX9 protein expression in BM samples from healthy controls (HC) and patients with aplastic anemia (AA).

(D) PCR Genotyping.

(E) qRT-PCR analysis to evaluate the deletion efficiency of *Dhx9* at the mRNA level. qRT-PCR primer pairs are designed and used for qRT-PCR analysis (n = 4).

(F) Western blot analysis of thymus, spleen and BM cells to confirm the DHX9 knockout efficiency at the protein level.

(G) Representative images showing smaller thymus and pale kidney from *Dhx9*$^{f/f;Vav1}$ mice (n = 3).

(H) Blood counts. White blood cell (WBC), red blood cell (RBC) and platelet (PLT) counts, hemoglobin (HGB), hematocrit (HCT) and mean corpuscular volume (MCV) were assessed within 2 weeks in control and *Dhx9* cKO mice (n = 10).

All mice used in the experiments were 7–14 days old. The data are presented as the mean ± SEM. ***P < 0.001; ****P < 0.0001; ns, not statistically significant; unpaired two-tailed Student's t-test.

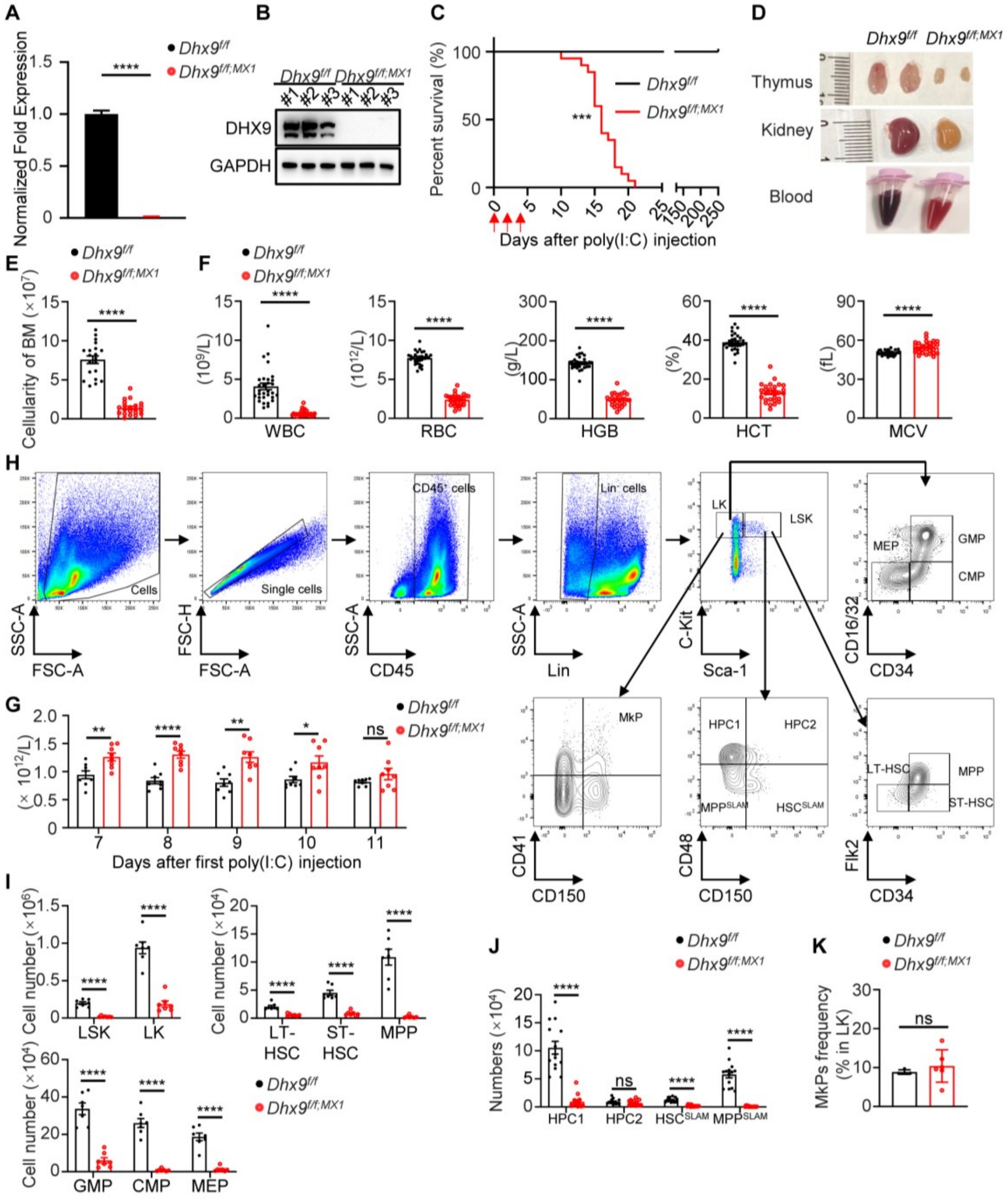

Figure S2. *Dhx9*$^{f/f;MX1}$ mice have the similar phenotypes to *Dhx9*$^{f/f;Vav1}$ mice, related to Figure 2.
(A) qRT-PCR analysis of *Dhx9* expression in BM cells from *Dhx9*$^{f/f}$ and *Dhx9*$^{f/f;MX1}$ mice (n = 4).
(B) Western blot analysis of DHX9 expression in BM cells from *Dhx9*$^{f/f}$ and *Dhx9*$^{f/f;MX1}$ mice (n = 3); GAPDH was used as a loading control.
(C) Kaplan–Meier survival curve of mice after poly(I:C)-induced *Dhx9* deletion, with red arrows marking the time points of poly(I:C) administration (n = 20).
(D) Representative images showing smaller thymus, pale kidney and blood from *Dhx9* cKO mice.
(E) Whole bone-marrow cellularity of *Dhx9*$^{f/f}$ (n = 20) and *Dhx9*$^{f/f;MX1}$ mice (n = 21).
(F) The routine blood parameters of *Dhx9*$^{f/f}$ (n = 30) and *Dhx9*$^{f/f;MX1}$ (n = 29) mice.
(G) Starting seven days after the first poly(I:C) injection, peripheral blood platelet counts were measured daily for five consecutive days (n = 8).
(H) Representative gating strategies used in FACS analyses for the frequencies of HSCs, LSK cells, and indicated lineage progenitors in mice.
(I and J) Absolute numbers of different hematopoietic stem and progenitor cell populations in the bone marrow from *Dhx9*$^{f/f}$ and *Dhx9*$^{f/f;MX1}$ mice (n ≥ 7).
(K) The proportion of $CD41^+CD150^+$ cells within the LK population was analyzed on day 11 following the first poly(I:C) injection in *Dhx9*$^{f/f}$ (n = 4) and *Dhx9*$^{f/f;MX1}$ mice (n = 6).
All mice used in these experiments except (K) were analyzed seven days after the first poly(I:C) injection. The data are presented as the mean ± SEM. *$P < 0.05$; **$P < 0.01$; ***$P < 0.001$; ****$P < 0.0001$; ns, not statistically significant; statistical significance was determined using the log-rank test for (C) and unpaired two-tailed Student's *t*-test for (A), (E-G) and (I-K).

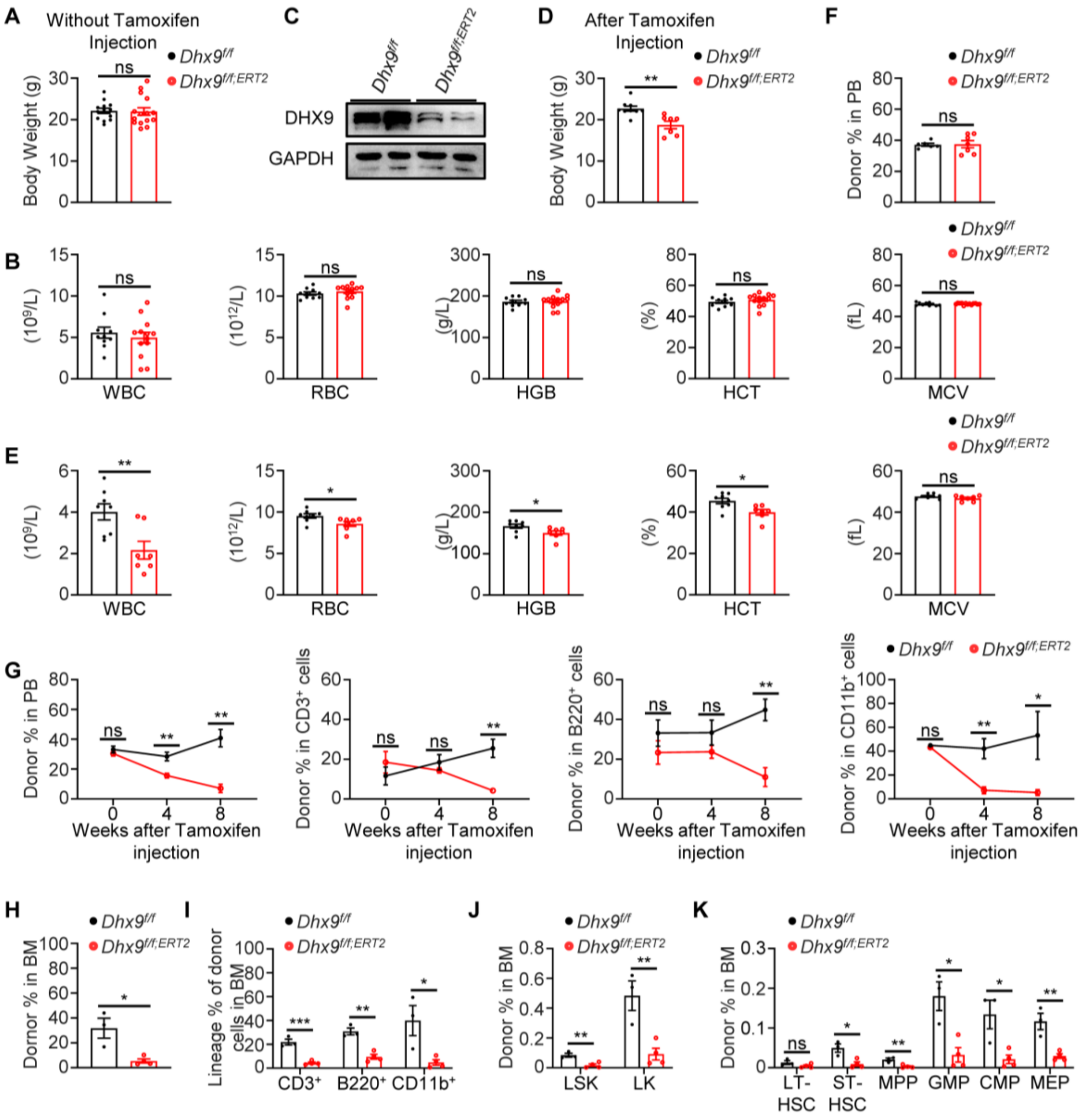

Figure S3. DHX9 sustains HSC for long-term hematopoiesis, related to Figure 3.

(A) Body weights of *Dhx9*$^{f/f}$ (n = 13) and *Dhx9*$^{f/f;ERT2}$ (n = 15) mice without tamoxifen injection.

(B) PB analysis of *Dhx9*$^{f/f}$ (n = 10) and *Dhx9*$^{f/f;ERT2}$ (n = 13) mice without tamoxifen injection.

(C) Western blot analysis of DHX9 expression in BM cells from *Dhx9*$^{f/f}$ and *Dhx9*$^{f/f;ERT2}$ mice after tamoxifen injection.

(D) Body weights of *Dhx9*$^{f/f}$ (n = 9) and *Dhx9*$^{f/f;ERT2}$ (n = 7) mice after tamoxifen injection.

(E) PB analysis of *Dhx9*$^{f/f}$ (n = 8) and *Dhx9*$^{f/f;ERT2}$ (n = 7) mice after tamoxifen injection.

(F) Donor chimerism in the PB of recipient mice was measured at four weeks after BMT without tamoxifen injection, and the results are graphed. *Dhx9*$^{f/f}$ (n = 6) and *Dhx9*$^{f/f;ERT2}$ (n = 7).

(G to K) The contributions of CD45.2^{+} donor-derived cells in total CD45^{+}, T (CD3^{+}), B (B220^{+}) and myeloid (CD11b^{+}) cells from PB (G) or BM (H, I) and LSK, LK, HSC and other hematopoietic populations in the BM (J, K) were analyzed in primary recipients after tamoxifen injection. *Dhx9*$^{f/f}$ (n = 3) and *Dhx9*$^{f/f;ERT2}$ (n = 4).

The mice shown in (C, D and E) were used two weeks after the first tamoxifen injection. The data are presented as the mean ± SEM. **P* < 0.05; ***P* < 0.01; ****P* < 0.001; ns, not statistically significant; unpaired two-tailed Student's *t*-test.

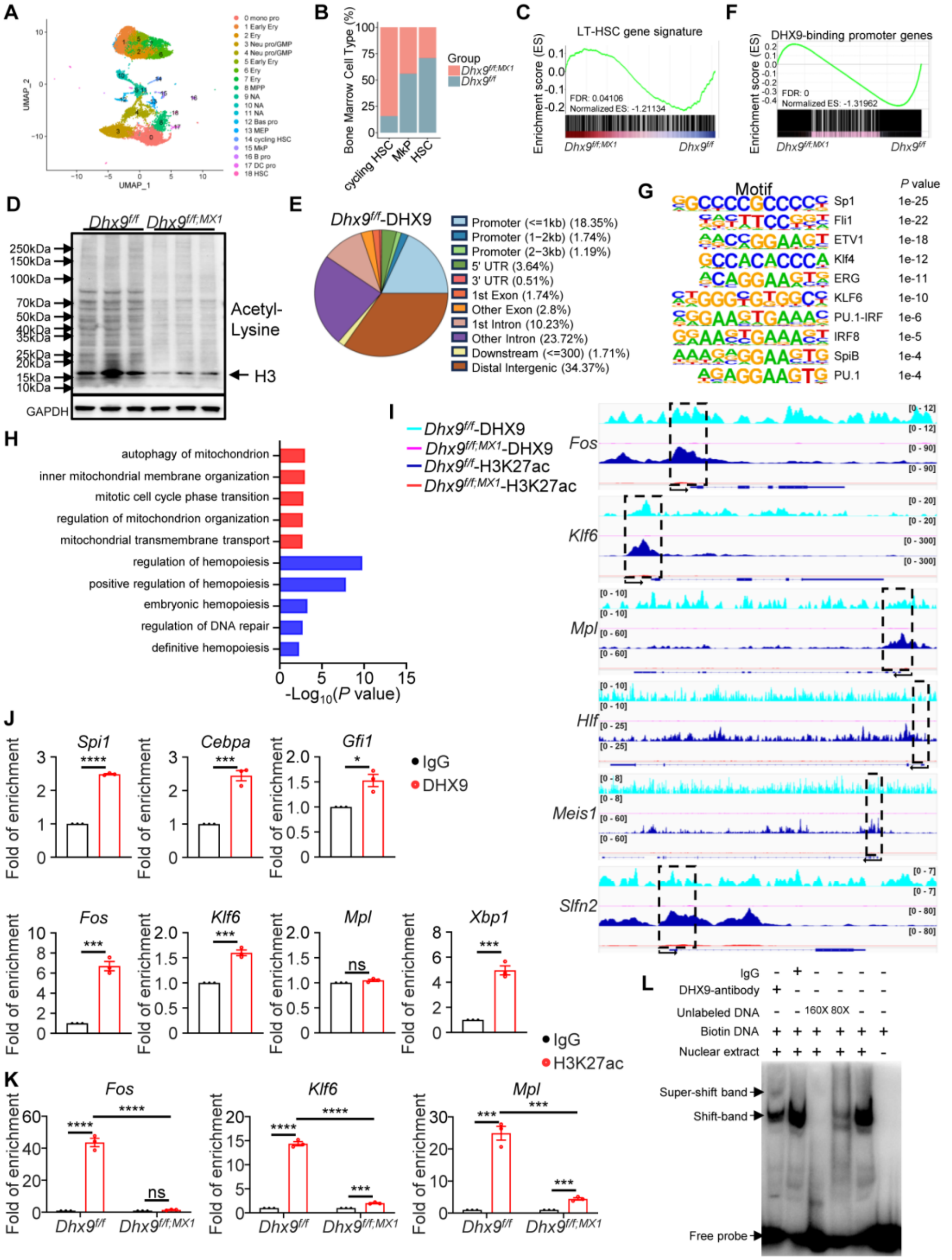

Figure S4. DHX9 promotes histone acetylation for transcriptional activation, related to Figure 5. (A) UMAP representation of scRNA-seq data from combined Lin$^-$c-Kit$^+$ cells from BM of *Dhx9$^{f/f}$* and *Dhx9$^{f/f;MX1}$* mice.

(B) Proportion of *Dhx9*$^{f/f}$ and *Dhx9*$^{f/f;MX1}$ cells within each annotated cluster.
(C) GSEA was performed using genes ranked by $\log_2$ fold change between *Dhx9*$^{f/f;MX1}$ and *Dhx9*$^{f/f}$ HSC cluster cells. ES, enrichment score; FDR, false discovery rate.
(D) Western blot analyses of pan-lysine acetylation in TNCs from BM of Dhx9$^{f/f}$ and *Dhx9*$^{f/f;MX1}$ mice.
(E) Pie chart showing genomic annotation of DHX9-associated peaks around genes.
(F) Leading edge plot showing the enrichment of genes associated with DHX9-binding promoter genes in the RNA-seq data from *Dhx9*$^{f/f}$ and *Dhx9*$^{f/f;MX1}$ BM Lin$^-$ cells. ES, enrichment score; FDR, false discovery rate.
(G) Transcription factor motifs analysis enriched in DHX9-enriched promoter peak genes.
(H) Pathway analysis of genes associated with H3K27ac downregulated (blue) or upregulated (red) regions in *Dhx9*$^{f/f;MX1}$ Lin$^-$ cells.
(I) IGV genome browser tracks showing ChIP-seq data for DHX9 (*Dhx9*$^{f/f}$ Lin$^-$ cells)–, DHX9 (*Dhx9*$^{f/f;MX1}$ Lin$^-$ cells)–, H3K27ac (*Dhx9*$^{f/f}$ Lin$^-$ cells)–, and H3K27ac (*Dhx9*$^{f/f;MX1}$ Lin$^-$ cells)–bound promoter regions of hematopoiesis-related genes.
(J) ChIP-qPCR analysis of DHX9–bound sites in hematopoiesis-related gene promoters in *Dhx9*$^{f/f}$ Lin$^-$ cells (n = 3).
(K) ChIP-qPCR analysis of H3K27ac–bound sites in hematopoiesis-related gene promoters with or without *Dhx9* (n = 3).
(L) Super-shift electrophoretic mobility shift assay (EMSA) for the analysis of DHX9 binding on Xbp1 promoter region (n = 3), with data obtained from three independent experiments.
The data are presented as the mean ± SEM. **P* < 0.05; ****P* < 0.001; *****P* < 0.0001; ns, not statistically significant; unpaired two-tailed Student's *t*-test.

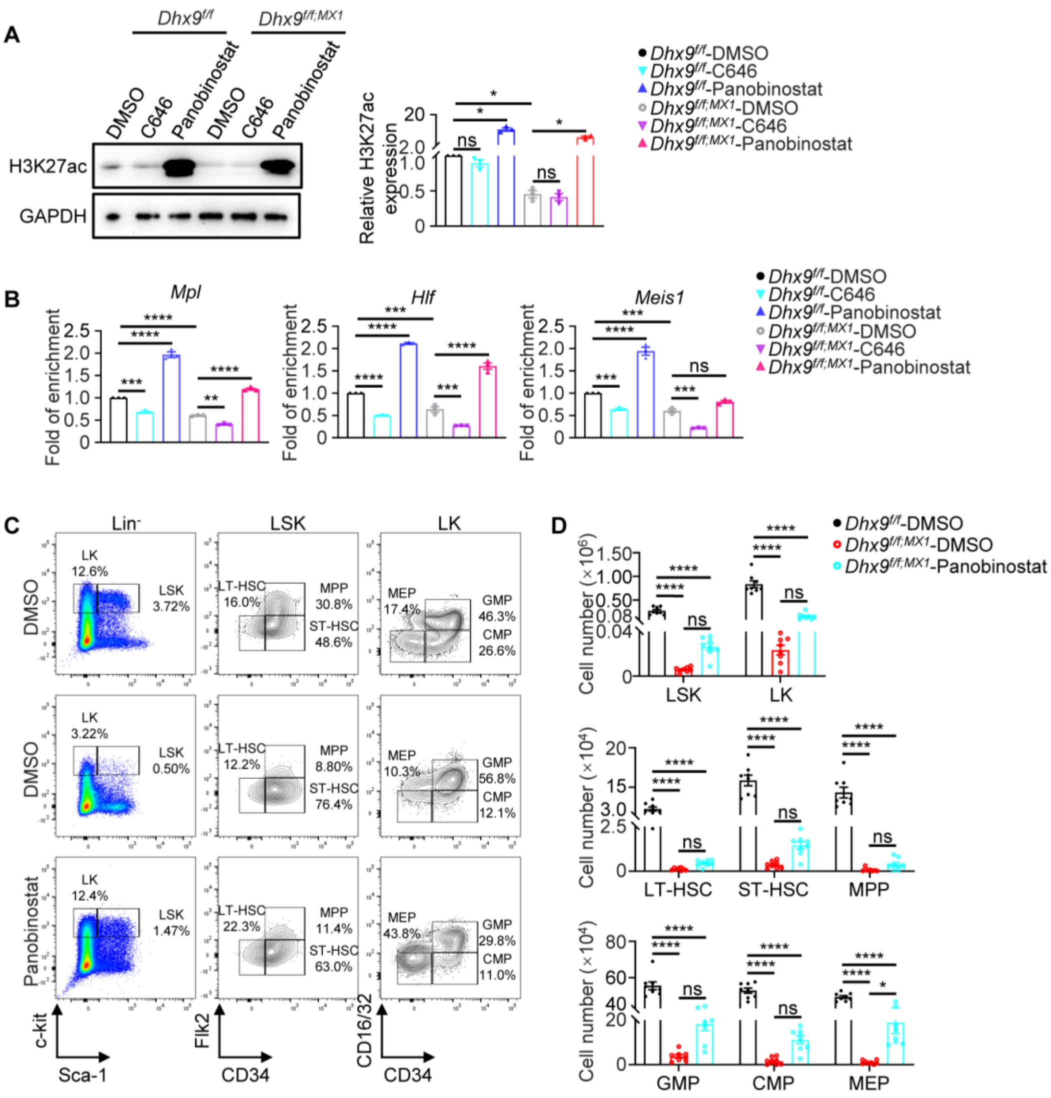

Figure S5. Increasing the level of H3K27ac partially rescue *Dhx9*-deficient HSCs in mice, related to Figure 5.

(A) BM cells isolated from *Dhx9$^{f/f}$* or *Dhx9$^{f/f;MX1}$* mice were treated with 40 μM C646 or 20 nM panobinostat for 48 h, then cells were lysed and subjected to immunoblotting analysis (left panel, n=3). The right panel shows the quantitative data of H3K27ac signal from three independent experiments.

(B) qRT-PCR analysis of hematopoiesis-related genes in *Dhx9$^{f/f}$* or *Dhx9$^{f/f;MX1}$* hematopoietic cells after treated with C646 or panobinostat (n = 3).

(C) Representative gating strategies used in FACS analyses for the frequencies of HSCs, LSK cells, and indicated lineage progenitors in mice following DMSO or 5 mg/kg panobinostat treatment.

(D) Absolute numbers of HSPC populations in the bone marrow of DMSO- or 5 mg/kg panobinostat-treated mice (n = 8).

The data are presented as the mean ± SEM. **P* < 0.05; ***P* < 0.01; ****P* < 0.001; *****P* < 0.0001; ns, not statistically significant; one-way ANOVA with Tukey's test (A, B and D).

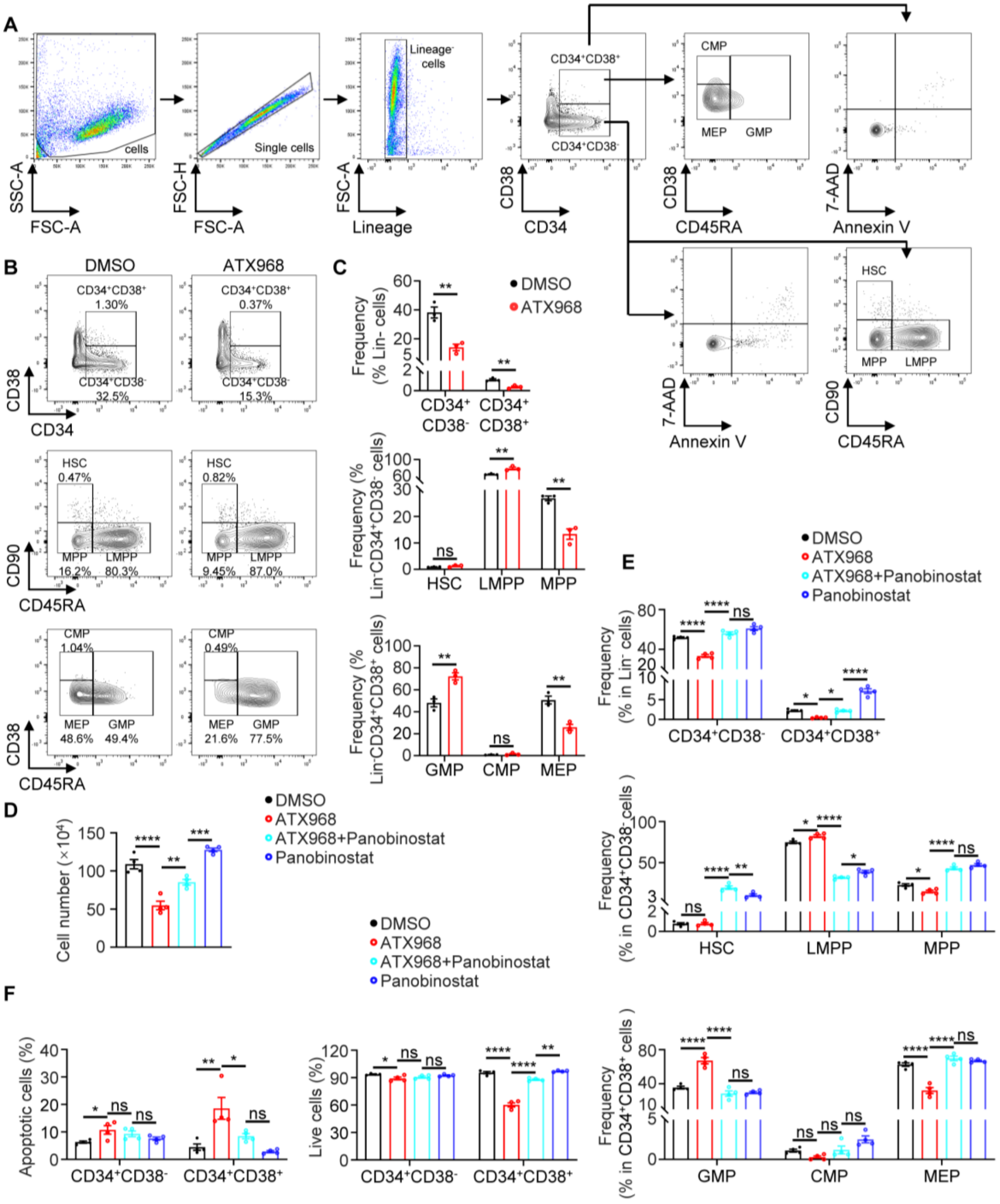

Figure S6. Inhibition of DHX9 in umbilical cord blood compromises HSC survival, related to Figure 6.

(A) Representative FACS gating strategies used to analyze the frequencies of HSCs and indicated lineage progenitors in human UCB cells.

(B and C) Representative FACS plots (B) and frequencies (C) of HSCs and indicated lineage progenitors in UCB CD34$^+$ cells following 6-day treatment with DMSO or ATX968 (n = 3).

(D) Total cell counts in UCB CD34$^+$ cells following 6-day treatment with DMSO, 40 μM ATX968, and/or 1 nM panobinostat (n = 4).

(E) Frequencies of HSCs and indicated lineage progenitors in UCB $CD34^+$ cells following 6-day treatment with DMSO, 40 μM ATX968, and/or 1 nM panobinostat (n = 4).
(F) Apoptotic cell ratios of $CD34^+CD38^-$ and $CD34^+CD38^+$ populations in UCB $CD34^+$ cells after 6-day treatment with DMSO, 40 μM ATX968, and/or 1 nM panobinostat (n = 4).
The data are presented as the mean ± SEM. *$P < 0.05$; **$P < 0.01$; ***$P < 0.001$; ****$P < 0.0001$; ns, not statistically significant; unpaired two-tailed Student's *t*-test (C), one-way ANOVA with Tukey's test (D-F).

Table S1. List of qRT-PCR primers used in this study.

	Forward	Reverse
Gapdh	CTTCATTGACCTCAACTACATGG	CTCGCTCCTGGAAGATGGTGAT
Dhx9	GTTCTTTCGCCGTTCTCGTG	GGTGTCTTCTTCACACCCTCC
Spi1	GACCAGGAACGAGAGCCATC	TCTGCAGCTCTGTGAAGTGG
Cebpa	CAAGAACAGCAACGAGTACCG	GTCACTGGTCAACTCCAGCAC
Gfi1	CCCTTTGCGTGCGAGATGT	CAGCGTGGATGACCTCTTGAA
Fos	TTTCAACGCCGACTACGAGG	TCTGCGCAAAAGTCCTGTGT
Hlf	TTCCCGATGATTTGAAGCAGGA	CCGGATTGCGATCTGGTTCT
Meis1	AAGGTGATGGCTTGGACAAC	TGTGCCAACTGCTTTTTCTG
Xbp1	TCCGCAGCACTCAGACTATG	TGCCCAAAAGGATATCAGACTCA
Klf6	GAAAGCACATCAGCGCACTC	GACCTGGAGAAACACCTGTCA
Mpl	TTGTGGATAGTGTGGGCCTG	CCATAGCGGAGTTCATGCCT
Slfn2	AACCACAGAACTCAGAGGAATG	GCTCCGAGATTTAGACCCAGC

Table S2. List of ChIP-qPCR primers used in this study.

	Forward	Reverse
ChIP-*Spi1*	CTGCTTCGACTTCCCCCTTT	ACTCACCTCTCGTTCCTGGT
ChIP-*Cebpa*	GGTGGTGTCCCGAACACTTG	CAGGCCAGAGCGATAGGATT
ChIP-*Gfi1*	TCAGCTTCGTTGGGCGTATT	GTCCGCAGAAAGAAACCTGC
ChIP-*Fos*	AATCCTACACGCGGAAGGTC	GCGCTCTGTCGTCAACTCTA
ChIP-*Xbp1*	GCCTTCACGGATATCGCTCT	CCCGTTGCGTCATATGCTTC
ChIP-*Klf6*	CCGAGTTCGAATTGCACCAA	ATGTCAGCTCGCACCAATCG
ChIP-*Mpl*	ACACAGTGCCGGAGAAGATG	TGGTGACTTGTGCCTGGTTT

Table S3. Key resources table.

REAGENT OR RESOURCE	SOURCE	IDENTIFIER
Antibodies		
Rabbit monoclonal anti-GAPDH	Cell Signaling Technology	Cat# 2118; RRID:AB_561053
Rabbit polyclonal anti-DHX9	Abcam	Cat# ab26271; RRID:AB_777725
Rabbit polyclonal anti-DHX9	Abcam	Cat# ab70777; RRID:AB_1270326
Rabbit monoclonal anti-H3K9ac	Cell Signaling Technology	Cat# 9649; RRID:AB_823528
Rabbit polyclonal anti-H3K27ac	Abcam	Cat# ab4729; RRID:AB_2118291
Rabbit monoclonal anti-IgG	Cell Signaling Technology	Cat# 3900; RRID:AB_1550038
Rabbit monoclonal anti-HA	Cell Signaling Technology	Cat# 3724; RRID:AB_1549585
Rabbit monoclonal anti-CBP	Cell Signaling Technology	Cat# 7389; RRID:AB_2616020
Mouse monoclonal anti-p300	Santa Cruz Biotechnology	Cat# sc-32244; RRID:AB_628076
Alexa Fluor™ Plus 647 anti-rabbit IgG	Thermo Fisher Scientific	Cat# A32733; RRID:AB_2633282
Alexa Fluor™ 488 anti-mouse IgG	Thermo Fisher Scientific	Cat# A11029; RRID:AB_2534088
DAPI Solution	BD Biosciences	Cat# 564907; RRID:AB_2869624
FITC anti-mouse Lineage Cocktail	BioLegend	Cat# 133301; RRID: AB_10697030
Brilliant Violet 510™ anti-mouse CD45	BioLegend	Cat# 103138; RRID:AB_312973
PE/Cyanine7 anti-mouse CD45.1	BioLegend	Cat# 110730; RRID:AB_1134168
Alexa Fluor® 700 anti-mouse CD45.2	BioLegend	Cat# 109822; RRID:AB_493731
APC anti-mouse c-kit	BioLegend	Cat# 105812; RRID:AB_313221
PE/Cyanine7 anti-mouse c-kit	BD Biosciences	Cat# 558163; RRID:AB_647250
Pacific Blue™ anti-mouse Sca-1	BioLegend	Cat# 122520; RRID:AB_2143237
Alexa Fluor 700 anti-mouse CD34	eBioscience	Cat# 56-0341-82; RRID: AB_493998
PE-CF594 anti-Mouse Flk2	BD Biosciences	Cat# 562537; RRID: AB_2737639
PE/Cy7 anti-mouse CD150	BioLegend	Cat# 115914; RRID:AB_439797
FITC anti-mouse CD48	BioLegend	Cat# 103404; RRID:AB_313019
APC anti-mouse CD48	BioLegend	Cat# 103411; RRID: AB_571996
FITC anti-mouse CD16/32	BioLegend	Cat# 101306; RRID:AB_312805
PE anti-mouse B220	BioLegend	Cat# 103208; RRID:AB_312993
PE anti-mouse CD3ε	BioLegend	Cat# 100308; RRID:AB_312673
PE anti-mouse CD2	BioLegend	Cat# 100108; RRID:AB_2073690
PE anti-mouse Gr-1	BioLegend	Cat# 108408; RRID:AB_313373
PE anti-mouse Ter-119	BioLegend	Cat# 116208; RRID:AB_313709
PE anti-Mouse CD41	BD Biosciences	Cat# 558040; RRID:AB_397004
eFluor 450 anti-mouse CD3	eBioscience	Cat# 48-0032-82; RRID: AB_1272193
FITC anti-mouse/human CD11b	BioLegend	Cat# 101206; RRID:AB_312789
Alexa Fluor® 488 anti-mouse/human Ki-67	BioLegend	Cat# 151204; RRID:AB_2566800
Alexa Fluor® 488 anti-H2A.X Phospho (Ser139)	BioLegend	Cat# 613405; RRID:AB_528914
Brilliant Violet 510™ anti-human Lineage Cocktail	BioLegend	Cat# 348807; RRID:AB_3097689
BV421 anti-human CD34	BD Biosciences	Cat# 562577; RRID:AB_2687922

Brilliant Violet 605™ anti-human CD90 (Thy1)	BioLegend	Cat# 328128; RRID:AB_2562281
APC anti-human CD38	eBioscience	Cat# 17-0389-42; RRID:AB_1834353
APC-eFluor™ 780 anti-human CD45RA	eBioscience	Cat# 47-0458-42; RRID:AB_10853641
Chemicals, peptides, and recombinant proteins		
ACK lysis buffer	Gibco	Cat# A1049201
TRIzol	Invitrogen	Cat# 15596018CN
Protease inhibitor cocktail	Sigma-Aldrich	Cat# 11873580001
DMSO	Sigma	Cat# D2650
poly (I:C)	Sigma	Cat# P1530
Tamoxifen	Sigma	Cat# T5648
C646	Sigma	Cat# 382113
Panobinostat	Selleckchem	Cat# LBH589
ATX968	TargetMol	Cat# T82568
Protein G Sepharose 4 Fast Flow	GE Health	Cat# 17-0618-01
StemSpan™ SFEM II	StemCell Technologies	Cat# 09655
Methocult GF M3434	StemCell Technologies	Cat# 03434
MethoCult™ H4435	StemCell Technologies	Cat# 04435
Murine SCF	Peprotech	Cat# 250-03
Murine IL3	Peprotech	Cat# 213-13
Murine FLT3L	Peprotech	Cat# 250-31L
Murine IL6	Peprotech	Cat# 216-16
Human SCF	Peprotech	Cat# 300-07-10UG
Human FLT3L	Peprotech	Cat# 300-19-10UG
Human TPO	Peprotech	Cat# 300-18-10UG
Human IL3	Peprotech	Cat# 200-03-10UG
Human IL6	Peprotech	Cat# 200-06-20UG
Fetal Bovine Serum	Bioind	Cat# 04-001-1A
Polyethylenimine	Polysciences	Cat# 23966
Critical commercial assays		
Human CD34 MicroBead Kit	Miltenyi Biotec	Cat# 130-046-702
Lineage Cell Depletion Kit mouse	Miltenyi Biotec	Cat# 130-090-858
CD117 MicroBeads, Miltenyi Biotec	Miltenyi Biotec	Cat# 130-091-224
FITC Annexin V Apoptosis Detection Kit with 7-AAD	BioLegend	Cat# 640922
Foxp3/Transcription Factor Staining Buffer Set	eBioscience	Cat# 00-5523
Taq Pro Universal SYBR qPCR Master Mix	Vazyme	Cat# Q712-03
PrimeScript™ RT Master Mix	Takara	Cat# RR036A
CellROX® Oxidative Stress Reagents	Invitrogen	Cat# C10444
Hyperactive Universal CUT&Tag Assay Kit for Illumina Pro	Vazyme	Cat# TD904-01
Immobilon Western Chemiluminescent HRP Substrate	Millipore	Cat# WBKLS0500
Pierce™ BCA Protein Assay Kits	Thermo Fisher	Cat# 23225

Supplemental methods

293T transient transfection

293T cells (HyCyte, TCH-C101) were cultured in DMEM supplemented with 10% FBS and 1% penicillin-streptomycin. The plasmids pLVX-*DHX9*-IRES-ZsGreen and pEnCMV-*CREBBP*-HA-SV40-Neo (MiaoLing Biology, P29740) were co-transfected using polyethylenimine.

Cell apoptosis, cell cycle and DNA damage staining

Briefly, for cell apoptosis analysis, stained HSPCs were stained for 15 min with the FITC-labeled Annexin V and 7-AAD Viability Staining Solution in Annexin V binding buffer. For nuclear protein staining, stained HSPCs were further fixed and permeabilized using a Cytofix/Cytoperm kit. For cell cycle analysis, cells were stained with an anti-Ki67 antibody and DAPI. For DNA damage analysis, cells were stained with anti-γH2A.X antibody.

Determination of reactive oxygen species (ROS) generation

Briefly, BM cells from WT or $Dhx9^{f/f;MX1}$ mice were harvested and stained with HSPCs markers, including lineage cocktail, antibodies against CD45, Sca-1, CD34, Flk2, c-Kit. For analysis of ROS, cells were stained with 5 μM CellRox in 1× Phosphate buffer saline (PBS) at 37°C for 30 minutes, and then resuspended in warm PBS to assess the ROS intermediate levels.

Colony-forming unit assay

Freshly isolated mouse $Lin^-c\text{-}Kit^+$ cells (2×10^3 cells) and human UCB $CD34^+$ cells (4×10^4 cells) were suspended in 100 μL IMDM supplemented with 2% FBS and plated in 1 mL of methylcellulose-based medium. Mouse BM cells were cultured in MethoCult™ GF M3434, while UCB $CD34^+$ cells were cultured in MethoCult™ GF H4435 with either DMSO or ATX968 (40 μM). All samples were cultured in triplicate and incubated at 37°C with 5% CO_2 and ≥ 95% humidity for 12 days. Hematopoietic colonies were counted under an inverted microscope. For both mouse and UCB samples, the mean of triplicates was used for final statistical analyses.

Confocal imaging

Lin$^-$ BM cells were placed on glass slides. The cells were fixed with 4% paraformaldehyde for 15 min at RT, washed with PBS and then permeabilized with 0.3% Triton X-100 for 10 min. After being blocked with 5% BSA for 1 h at RT, cells were stained overnight at 4°C with primary antibodies, followed by incubation with secondary antibodies for 1 h at RT. The primary antibodies were anti-DHX9, anti-p300, anti-H3K27ac. The secondary antibodies were goat anti-mouse Alexa Fluor™ 488 and/or goat anti-rabbit Alexa Fluor™ Plus 647. After washing, cells mounted with DAPI. Fluorescence was examined using a confocal microscope (Zeiss, LSM800) and staining intensity was quantified by ImageJ program.

Primary cell culture and chemical treatment

In mice, total BM cells were harvested and cultured in HSC expansion media consisting of StemSpan SFEM supplemented with murine 50 ng/ml SCF, 20 ng/ml Flt-3 ligand, 10 ng/ml IL-3, and 10 ng/ml IL-6 after a quick RBC lysis. C646 and the HDACi panobinostat were used in BM cells at 40 μM and 20 nM respectively.

All umbilical cord blood units used were obtained under the approval of the Ethics Committee of the First Affiliated Hospital of USTC (2023-RE-388). Human UCB CD34$^+$ cells were isolated using the human CD34 MicroBead Kit. These cells were cultured in HSC expansion media consisting of StemSpan SFEM supplemented with human 100 ng/ml SCF, 100 ng/ml Flt-3 ligand, 50 ng/ml thrombopoietin, 10 ng/ml IL-3, and 10 ng/ml IL-6. DHX9 specific inhibitor ATX968 and HDACi panobinostat were used in CD34$^+$ cord blood cells at 40 μM and 1 nM respectively.

Protein extraction and immunoblot analysis

Human BM samples were obtained under the approval of the Ethics Committee of the First Affiliated Hospital of USTC (2023-RE-307). Cells were harvested by centrifugation at 500 × g for 5 min, washed with cold PBS and lysed in NP-40 lysis buffer supplemented with 1% protease inhibitor cocktail on ice for 30 min, then centrifuged at 12000 × g for 15 min at 4°C to harvest the supernatant. Protein concentration was measured using a Nanophotometer (IMPLEN, NP80) with a BCA protein assay kit. Furthermore, equal amounts of protein extracts were fractionated on 4%~12% gradient SDS–PAGE gels and transferred to PVDF membranes. The blots were blocked with 5% nonfat milk at RT for 1 hour and then incubated with

primary antibodies overnight at 4°C. After incubation, membranes were washed with TBST, and then incubated with secondary antibody for 1 h at RT. Bands were captured using the FluorChem M system (ProteinSimple, USA).

For immunoprecipitation, 293T cells were collected at 48 h after transfection using lysis buffer [containing 50 mM Tris-HCl, (pH 7.4), 150 mM NaCl, 2 mM EDTA, 5% glycerol, and 0.5% NP-40] supplemented with PMSF and a protease inhibitor cocktail. The lysates were diluted to approximately 1 mg/ml with lysis buffer, 500 μg of the total cell lysate was incubated with the indicated antibody overnight at 4°C, protein A/G-Sepharose beads were added, and the mixture was further incubated for 1.5 h at 4°C. After centrifugation at 12000 × g for 30 s, the beads were washed 6 times with cell lysis buffer before analysis. The primary antibodies used in this study including anti-GAPDH, anti-DHX9, anti-H3K27ac, anti-H3K9ac, anti-HA, anti-CBP and anti-IgG.

RNA isolation and quantitative reverse transcription PCR (qRT-PCR) analysis

Total RNA was isolated using TRIzol reagent. The cDNA was synthesized using the PrimeScript™ RT Master Mix and subjected to qPCR with SYBR Green qPCR Master Mix. All primers used are listed in Table S1. *Gapdh* was used as an internal normalization control.

CUT&Tag assay and ChIP-qPCR analysis

CUT&Tag assays were performed according to the manufacturer's instructions. Briefly, prewashed Lin$^-$ cells were incubated with concanavalin A–coated magnetic beads, incubated with anti-DHX9, anti-H3K27ac, or normal anti-IgG primary antibodies for overnight at 4°C and incubated secondary antibodies at room temperature (RT) for 1 h. After three washes, the samples were incubated with pA/G-Tnp Pro at RT for 1 h. The extracted DNA fragments were subjected to PCR amplification, and then performed high-throughput sequencing. Clean reads were aligned to the mouse genome (GRCm39) via software Bowtie2 (version 2.2.7), and peak calling was done by using MACS3 (version 3.0.0a6). Different peaks were analyzed using edgeR (version 4.0.1) with a false discovery rate (FDR) < 0.05 and an absolute fold change ≥ 2. All libraries were sequenced on the Illumina Novaseq 6000 platform. ChIP-qPCR primers used in this study are listed in Table S2.

Super-shift electrophoretic mobility shift assay (EMSA)

Nuclear proteins (20 μg) were preincubated in the binding buffer with or without molar excess of unlabeled competitor double-stranded oligonucleotides or anti-DHX9 antibody at room temperature for 10 min. The 5′ biotin-TEG–labeled double-stranded DNA (dsDNA) probe corresponding to the XBP1 promoter region (5' - GAACAGCTGTGCAGCCACGCTGGACA - 3') was added, and the incubation was continued for 30 min. DNA–protein complexes were separated from free probes on a 6% native polyacrylamide gel at low temperature and detected by fluorescence imaging to assess binding specificity.

Single-cell (sc) RNA-seq, data preprocessing, and dimensionality reduction and data analysis

Freshly isolated mouse $Lin^{-}c\text{-}Kit^{+}$ cells with >90% viability were used to generate scRNA-seq libraries using the 10x Genomics Chromium Single Cell 3′ v3 kit, followed by sequencing on an Illumina NovaSeq 6000 platform. The raw sequencing reads were de-multiplexed and aligned to the mouse genome (GRCm39) using Cell Ranger (v6.0.2). The resulting gene-cell expression matrices were imported into R (v4.5.1) for downstream analysis using the *Seurat* (v4.3.0) and *SeuratObject* (v4.1.3) packages. Cells expressing fewer than three genes, showing high mitochondrial gene content, or identified as erythrocytes or doublets were removed. Gene expression matrices were normalized and scaled, and highly variable genes were identified for subsequent analyses. Principal component analysis (PCA) was then performed to capture major sources of biological variation, followed by clustering and visualization using the Uniform Manifold Approximation and Projection (UMAP) algorithm. Unsupervised cell clusters were annotated using the *scCATCH* package (v3.3.2), based on reference gene signatures and manually curated marker genes from previous studies (Dahlin et al., 2018; Kucinski et al., 2024; Poscablo et al., 2024; Rodriguez-Fraticelli et al., 2020).

Supplemental references

Dahlin, J.S., Hamey, F.K., Pijuan-Sala, B., Shepherd, M., Lau, W.W.Y., Nestorowa, S., Weinreb, C., Wolock, S., Hannah, R., Diamanti, E., et al. (2018). A single-cell hematopoietic landscape resolves 8 lineage trajectories and defects in Kit mutant mice. Blood 131, e1-e11. 10.1182/blood-2017-12-821413.

Kucinski, I., Campos, J., Barile, M., Severi, F., Bohin, N., Moreira, P.N., Allen, L., Lawson, H., Haltalli, M.L.R., Kinston, S.J., et al. (2024). A time- and single-cell-resolved model of murine bone marrow hematopoiesis. Cell Stem Cell 31, 244-259.e210. 10.1016/j.stem.2023.12.001.

Poscablo, D.M., Worthington, A.K., Smith-Berdan, S., Rommel, M.G.E., Manso, B.A., Adili, R., Mok, L., Reggiardo, R.E., Cool, T., Mogharrab, R., et al. (2024). An age-progressive platelet differentiation path from hematopoietic stem cells causes exacerbated thrombosis. Cell 187, 3090-3107.e3021. 10.1016/j.cell.2024.04.018.

Rodriguez-Fraticelli, A.E., Weinreb, C., Wang, S.W., Migueles, R.P., Jankovic, M., Usart, M., Klein, A.M., Lowell, S., and Camargo, F.D. (2020). Single-cell lineage tracing unveils a role for TCF15 in haematopoiesis. Nature 583, 585-589. 10.1038/s41586-020-2503-6.
