# Supplementary material for: DHX9 sustains hematopoietic stem cell function in cooperation with H3 acetylation
Source: Stem Cell Reports. 2026 Feb 5;21(3):102794. doi: 10.1016/j.stemcr.2026.102794 (PMC12985375; doi:10.1016/j.stemcr.2026.102794)
Supplement: Document S2. Article plus supplemental information [file mmc2.pdf]

STEM CELL REPORTS
Article
OPEN ACCESS

DHX9 sustains hematopoietic stem cell function in cooperation with H3 acetylation

Minhui Shi,[1,3,6] Mengqing Gao,[2,3,6] Huixin Luo,[1,3] Chong Wang,[1] Xueyang Hu,[1] Yacen Xiong,[1] Yan Chen,[1] Xingxing Ren,[4] Shu Zhu,[5] and Huaiping Zhu[1,3,7,*]
[1]Department of Clinical Laboratory, The First Affiliated Hospital of USTC, Division of Life Sciences and Medicine, University of Science and Technology of China, Hefei 230001, China
[2]Department of Hematology, The First Affiliated Hospital of USTC, Division of Life Sciences and Medicine, University of Science and Technology of China, Hefei 230001, China
[3]Blood and Cell Therapy Institute, Anhui Provincial Key Laboratory of Blood Research and Applications, University of Science and Technology of China, Hefei 230027, China
[4]Zhejiang University Center for Veterinary Sciences, College of Animal Sciences, Zhejiang University, Hangzhou 310058, China
[5]Institute of Immunology and the CAS Key Laboratory of Innate Immunity and Chronic Disease, Division of Life Sciences and Medicine, University of Science and Technology of China, Hefei 230027, China
[6]These authors contributed equally
[7]Lead contact
*Correspondence: huaipingzhu@ustc.edu.cn
https://doi.org/10.1016/j.stemcr.2026.102794

SUMMARY

Hematopoietic stem cells (HSCs) self-renew to sustain stem cell pools and differentiate into all types of blood cells, whose properties are tightly regulated by epigenetic and transcriptional networks. Here, we identified DHX9 as a critical regulator of HSC maintenance. *Dhx9* deletion caused bone marrow failure and impaired hematopoietic reconstitution in murine primary and secondary transplantation recipients due to loss of HSCs and defective self-renewal capacity. Further investigations revealed that *Dhx9* deficiency led to aberrant cell cycle entry, increased apoptosis, and elevated ROS, which compromise HSC function. Mechanistically, DHX9 interacts with CBP/p300 acetyltransferase and maintains H3 acetylation at hematopoietic gene promoters to facilitate transcription activation. Inhibition of CBP/p300 disrupted their expression, whereas the enhancement of H3K27ac levels partially rescued hematopoietic defects caused by *Dhx9* deficiency in both mouse models and human CD34$^+$ cells. This study highlights DHX9 as a crucial factor linking epigenetic modifications with transcriptional programs in HSC biology.

INTRODUCTION

Hematopoietic stem cells (HSCs) can renew themselves and differentiate into all lineages of blood cells throughout their lifetime (Boulais and Frenette, 2015). HSC function is tightly regulated by intrinsic mechanisms and extrinsic cues, which result in an intricate molecular state to balance self-renewal and differentiation (Mendelson and Frenette, 2014; Morrison and Scadden, 2014). Intrinsic factors involved in cellular metabolism, and epigenetic, or transcriptional regulation, have been reported to regulate HSC functions (Dzierzak and Bigas, 2018; Meng et al., 2023; Suda et al., 2011). Epigenetic mechanisms that dictate chromatin accessibility for transcription factors are crucial for cell fate decisions (Butler and Dent, 2013). Previous studies have shown that DNA methylation and histone modification dynamics, such as H3K27ac, H4K16ac, and H3K79me2, are required for HSC self-renewal and differentiation (Meng and Nerlov, 2025).

DEAH-box helicase 9 (DHX9), also known as RNA helicase A (RHA), a member of the DExD/H-box superfamily II of helicases, plays a central role in many cellular processes, including DNA replication, transcription, translation, RNA processing and transport, and maintenance of genomic stability (Ren et al., 2024). Global knockout of *Dhx9* is embryonic lethal in mice because of a differentiation defect in the embryonic ectoderm during gastrulation (Lee et al., 1998), and embryonic stem cells depleted of *Dhx9* are unable to differentiate (Leone et al., 2017), indicating that DHX9 is critical in early embryonic development. The RHA-1/L1TD1/LIN28 complex promotes the translation of the stem cell factor Oct4 by recruiting RHA-1 into the translation complex in human embryonic stem cells (hESCs) (Närvä et al., 2012), indicating a role for DHX9 in hESC renewal. These data indicate that DHX9 is essential during development. However, the role of DHX9 in HSC biology is unknown.

In this study, a critical role of DHX9 in HSC function was reported in mice with the conditional deletion of *Dhx9*. Deletion of *Dhx9* in hematopoietic cells led to hematopoietic failure of multilineage blood cells. Serial transplantation revealed that *Dhx9* deficiency resulted in impaired HSC reconstitution. We demonstrated that DHX9 promotes histone H3 acetylation at lysines 9 and 27 (K9, K27) at hematopoietic gene loci, possibly through interactions with CBP/p300. Genome-wide analysis via CUT&Tag revealed that H3K27ac signals at promoter regions of hematopoietic genes and regulators were decreased in *Dhx9* knockout cells, which is consistent with their decreased expression according to transcriptomic profiling. Notably,

© 2026 The Author(s).
Published by Elsevier Inc. on behalf of International Society for Stem Cell Research.
This is an open access article under the CC BY license (http://creativecommons.org/licenses/by/4.0/).

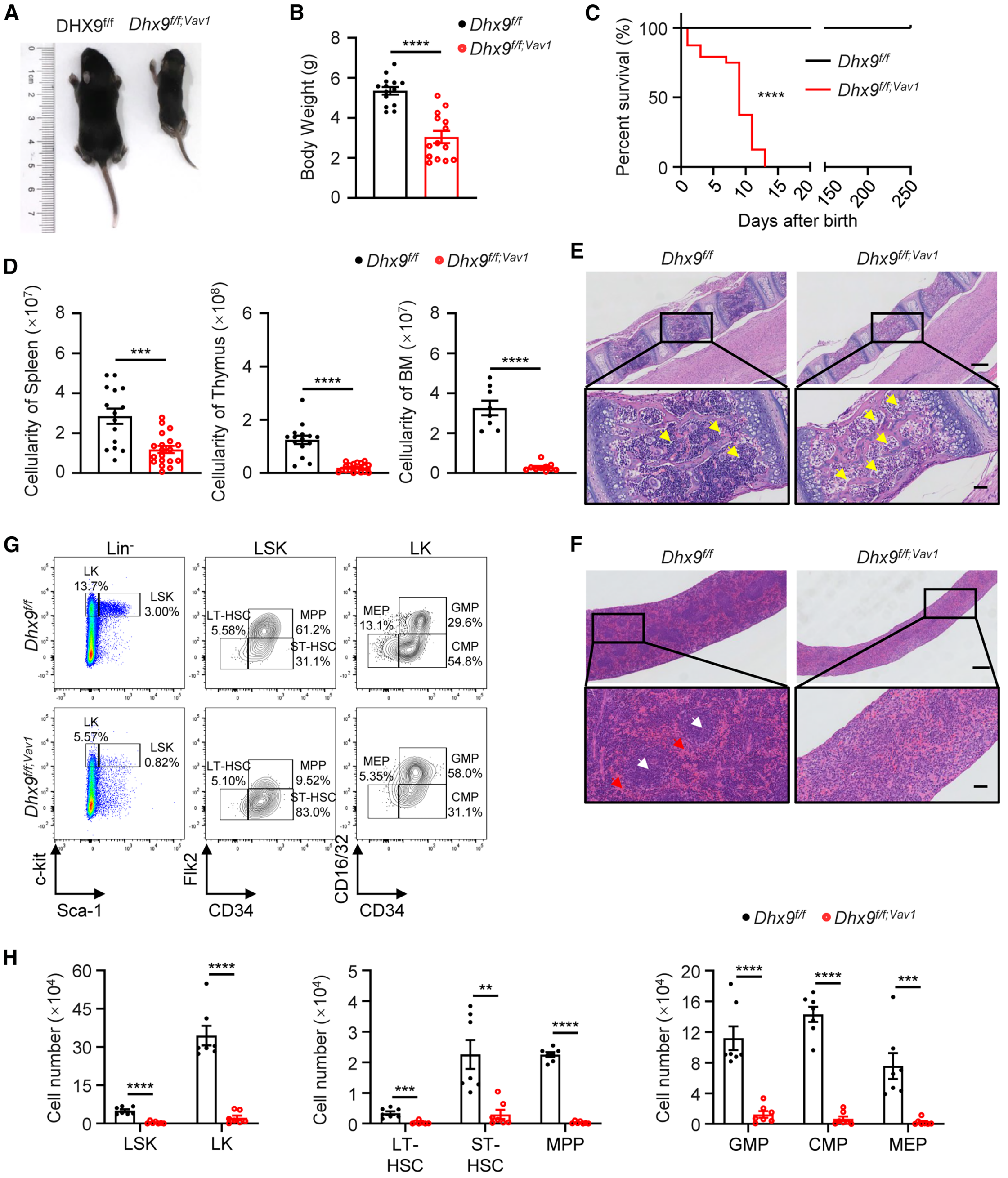

Figure 1. *Dhx9* deletion results in the lethality and depletion of hematopoietic stem cells

(A) A representative image of 12-day-old *Dhx9*$^{f/f}$ and *Dhx9*$^{f/f;Vav1}$ mice.

(B) Body weights of *Dhx9*$^{f/f}$ and *Dhx9*$^{f/f;Vav1}$ mice (n = 14).

(C) Kaplan–Meier survival curves of *Dhx9*$^{f/f}$ and *Dhx9*$^{f/f;Vav1}$ mice (n = 28).

(D) Total nucleated cell (TNC) numbers in the spleen (left), thymus (middle), and in the femurs and two tibias ($n \geq 8$).

(E and F) Representative hematoxylin and eosin (HE)-stained vertebrae (E) and spleens (F) from *Dhx9*$^{f/f}$ and *Dhx9*$^{f/f;Vav1}$ mice. Scale bars, 250 μm (top) and 50 μm (bottom). Yellow arrows, adipocytes; red arrows, red pulp; white arrows, white pulp.

(G) Representative fluorescence-activated cell sorting (FACS) analyses of LK cells, LSK cells, HSPCs, and lineage-restricted progenitors from *Dhx9*$^{f/f}$ and *Dhx9*$^{f/f;Vav1}$ mice.

(H) Absolute numbers of different HSPC populations from *Dhx9*$^{f/f}$ and *Dhx9*$^{f/f;Vav1}$ mice (n = 7).

(legend continued on next page)

these genes also overlapped with the binding peaks of DHX9. Our data suggest that DHX9-mediated gene transcription occurs through H3 acetylation, serving as a mechanism for controlling HSC self-renewal.

RESULTS

Genetic deletion of DEAH-box helicase 9 in hematopoietic cells leads to defective hematopoiesis

We first analyzed public datasets (GSE111085 and GSE16334) and found that *Dhx9* expression was markedly reduced in patients with myelodysplastic syndrome (MDS) and Fanconi anemia (FA). Consistently, clinical samples collected from patients with aplastic anemia (AA) also showed decreased DHX9 expression compared to those from healthy donors (Figures S1A–S1C), suggesting that DHX9 might be involved in the regulation of hematopoiesis. To explore the role of DHX9 in hematopoiesis, we generated mice with the specific deletion of *Dhx9* in hematopoietic cells ($Dhx9^{f/f;Vav1}$) by crossing $Dhx9^{f/f}$ (WT) (Ren et al., 2023) mice with Vav1-Cre mice (Figure S1D). We confirmed the effective deletion of DHX9 in splenocytes, thymocytes, and bone marrow (BM) cells in $Dhx9^{f/f;Vav1}$ mice (Figures S1E and S1F).

The $Dhx9^{f/f;Vav1}$ mice were undersized, accompanied by reduced thymus size and pale organ, and had a significantly shortened lifespan of approximately 2 weeks after birth (Figures 1A–1C and S1G). Complete blood count analysis showed significantly reduced white blood cells (WBCs), red blood cells (RBCs), hematocrit, and hemoglobin with an increased mean corpuscular volume (MCV). While there is no change in the levels of platelet (PLT) (Figure S1H). Reduced cellularity was observed in the spleen, thymus, and BM of $Dhx9^{f/f;Vav1}$ mice (Figure 1D). Histological analysis further showed that $Dhx9^{f/f;Vav1}$ mice had decreased BM cells, increased adipose infiltration in vertebrae (Figure 1E), and a disorganized structure of white and red pulp in the spleen (Figure 1F), suggesting defective hematopoiesis. We then examined the number of primitive hematopoietic stem and progenitor cells (HSPCs) in the BM of $Dhx9^{f/f}$ mice and $Dhx9^{f/f;Vav1}$ mice. Loss of *Dhx9* significantly reduced the absolute number of $Lin^{-}Sca\text{-}1^{+}c\text{-}Kit^{+}$ (LSK) cells, $Lin^{-}Sca\text{-}1^{-}c\text{-}Kit^{+}$ (LK) cells, long-term repopulating HSCs (LT-HSCs: $Flk2^{-}CD34^{-}LSK$), short-term repopulating HSCs (ST-HSCs: $Flk2^{-}CD34^{+}LSK$), multipotent progenitors (MPP: $Flk2^{+}$ $CD34^{+}LSK$), and fate-committed progenitors, including granulocyte-macrophage progenitors (GMPs: $CD16/32^{+}CD34^{+}LK$), common myeloid progenitors (CMPs: $CD16/32^{+}CD34^{-}LK$), and megakaryocyte-erythroid progenitors (MEPs: $CD16/32^{-}CD34^{-}LK$) (Figures 1G and 1H). Together, these data suggest that DHX9 is required for multilineage development in murine hematopoiesis.

Knockout of DEAH-box helicase 9 impairs hematopoietic stem cell survival

To determine the role of DHX9 in adult hematopoiesis, we generated $Dhx9^{f/f;MX1}$ (cKO) mice, in which Cre expression was induced by injecting polyinosine-polycytidylic acid [poly(I:C)], leading to the reduced expression of DHX9 in BM (Figures S2A and S2B). After poly(I:C) injection, $Dhx9^{f/f;MX1}$ mice ultimately resulted in lethality and recapitulated the phenotype of $Dhx9^{f/f;Vav1}$ mice, with reduced volume of thymus, pale kidneys and blood, and decreased BM cellularity (Figures S2C–S2E). Hematologic analysis further revealed similar alterations in both models, except that cKO mice showed a transient increase in PLT counts. A consecutive five-day assessment confirmed that PLT levels initially rose but subsequently returned to baseline, ultimately showing no significant difference from controls, consistent with the phenotype observed in $Dhx9^{f/f;Vav1}$ mice (Figures S2F and S2G).

Flow cytometric analysis was conducted to further assess the impact of *Dhx9* deletion on HSPC homeostasis (Figure S2H). Consistent with previous findings, a marked reduction in multiple HSPC subsets was observed following poly(I:C) administration in $Dhx9^{f/f;MX1}$ mice (Figure S2I). This decrease was further corroborated using an alternative gating strategy, which similarly revealed a significant decline in HSPC populations, including the HSC^{SLAM} population ($CD150^{+}CD48^{-}LSK$) and MPP^{SLAM} population ($CD150^{-}CD48^{-}LSK$) (Figure S2J), indicating that *Dhx9* deletion leads to HSC exhaustion. Interestingly, the proportion of $CD41^{+}CD150^{+}$ cells within the LK population remained comparable (Figure S2K).

To characterize the role of DHX9 in HSPCs, we sorted $Lin^{-}c\text{-}Kit^{+}$ cells and performed a colony-forming unit (CFU) assay *in vitro*. *Dhx9*-deficient cells showed decreased number and size of multipotential colony-forming units (CFU-GEMM), granulocyte-macrophage colony-forming units (CFU-GM), and erythroid burst-forming units (BFU-E) (Figures 2A and 2B). We next assessed DNA damage, apoptosis, and cell cycle in WT and *Dhx9*-deficient HSPCs. γH2A.X levels, a marker of DNA damage, were strongly increased in cKO HSPCs (Figures 2C and 2D). In addition, a significant increase in the frequency of apoptotic cells in the total BM, suggested that *Dhx9* deletion resulted in elevated apoptosis in HSPCs

All mice used in the experiments were 7–14 days old. The data are presented as the mean ± SEM. $^{**}p < 0.01$; $^{***}p < 0.001$; $^{****}p < 0.0001$; statistical significance was determined using the log rank test for (C) and unpaired two-tailed Student's *t* test for (B), (D) and (H). See also Figure S1.

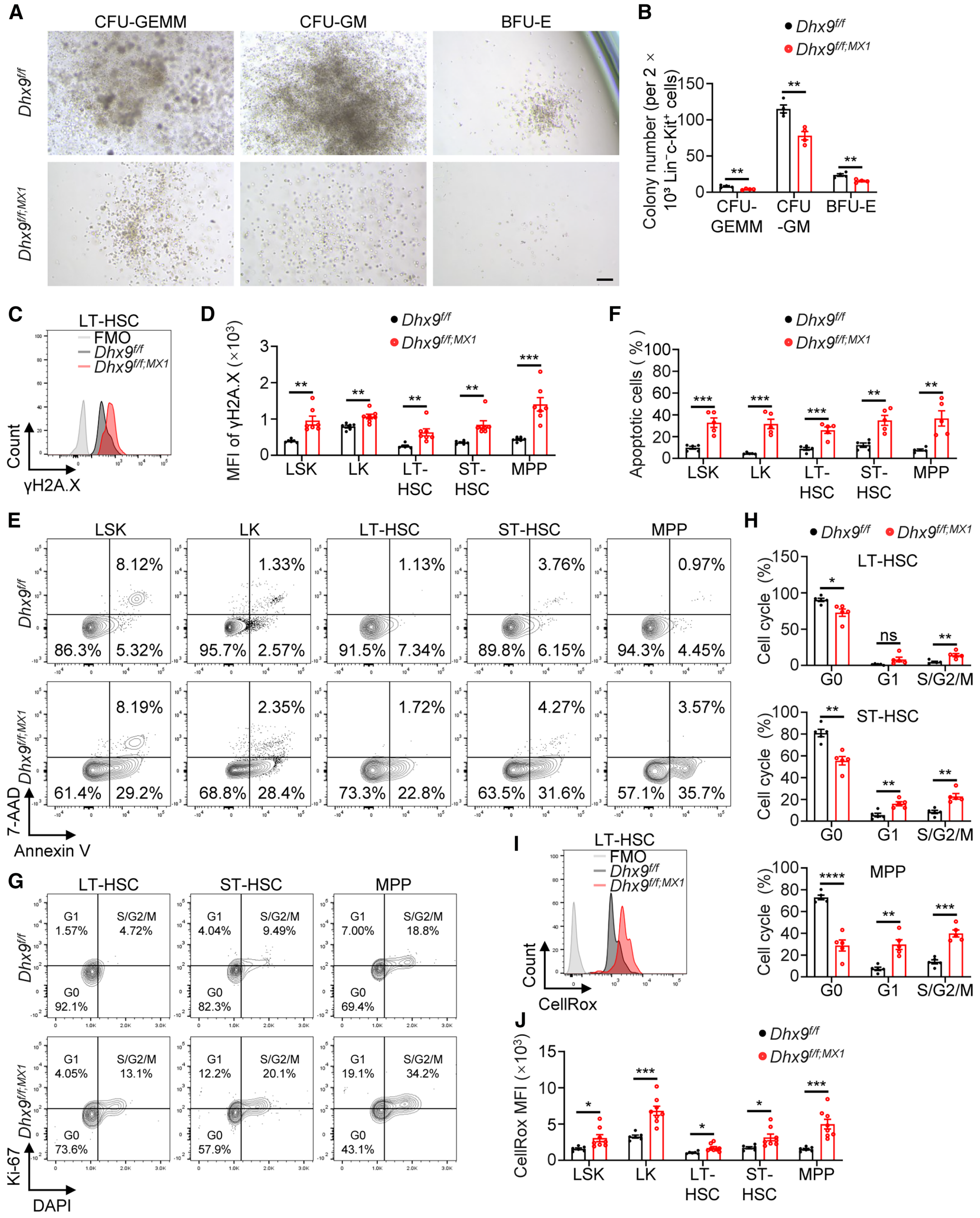

Figure 2. *Dhx9*-deficient HSCs have increased cycling and apoptosis

(A) Representative images of CFU assays of $Lin^-c\text{-}Kit^+$ cells from WT and cKO mice. Scale bars, 100 μm.

(B) Hematopoietic colonies were enumerated from methylcellulose cultures plated with 2×10^3 $Lin^-c\text{-}Kit^+$ cells ($n = 4$).

(C and D) Representative FACS analyses (C) and quantitation of γH2A.X mean fluorescence intensity (MFI) (D) in WT ($n = 6$) and cKO ($n = 7$) hematopoietic cells.

(E–H) Representative FACS plots show apoptosis (E) and cell cycle (G) in HSPCs. The percentages of apoptotic HSPCs (annexin V^+) (F) and HSPCs in the G0, G1, and S/G2/M phases (H) were quantified ($n = 5$).

(I and J) ROS levels in WT ($n = 6$) and cKO ($n = 8$) HSPCs were determined via CellRox Green.

(legend continued on next page)

(Figures 2E and 2F). Cell cycle analysis revealed an increased proportion of the S/G2/M phase in LT-HSCs, ST-HSCs and MPPs after *Dhx9* deletion (Figures 2G and 2H), indicating cell cycle entry of HSPCs, which could lead to the loss of quiescence and exhaustion of the HSPC pool. Given the pivotal role of low intracellular reactive oxygen species (ROS) levels in maintaining HSC quiescence (Hu et al., 2018; Singh et al., 2018), we assessed and found significantly increased ROS production in cKO mice (Figures 2I and 2J). These findings indicate that DHX9 is required for HSC survival and maintenance.

DEAH-box helicase 9 is required for hematopoietic stem cell reconstitution and self-renewal *in vivo*

We next determined the role of DHX9 in HSC reconstitution *in vivo*. Since homozygous knockout of *Dhx9* ($Dhx9^{f/f;Vav1}$ or $Dhx9^{f/f;MX1}$) causes rapid lethality due to hematopoietic failure, we performed a competitive BM transplantation (BMT) assay (Figure 3A), which can ensure the survival of recipient mice with severe defects caused by *Dhx9* deletion. Donor chimerism, represented by the total $CD45.2^+$ frequency in peripheral blood (PB), was assessed every 4 weeks after transplantation. Notably, the frequency of total chimerism from cKO mice was considerably lower at 4 weeks and did not recover 16 weeks after transplantation (Figure 3B). At the end of the experiment, BM chimerism was markedly reduced in $Dhx9^{f/f;MX1}$ recipients (Figure 3C). Donor-derived HSPC populations from $Dhx9^{f/f;MX1}$ mice also decreased robustly (Figure 3D), suggesting impaired HSC reconstitution.

To determine whether the *Dhx9* depletion-mediated HSC repopulation defects are cell-intrinsic and independent of deletion timing and HSC-homing ability, we introduced a $Dhx9^{f/f;ERT2}$ mouse model for BMT in which *Dhx9* deletion was induced by injecting tamoxifen following BMT (Figure 3E). Before tamoxifen injection, $Dhx9^{f/f;ERT2}$ mice had body weights and blood cell counts similar to controls (Figures S3A and S3B). However, tamoxifen administration resulted in reduced body weight and severe anemia, phenocopying $Dhx9^{f/f;MX1}$ mice (Figures S3C–S3E). Furthermore, BM cells isolated from $Dhx9^{f/f}$ and $Dhx9^{f/f;ERT2}$ mice were subsequently transplanted into lethally irradiated recipient mice, followed by tamoxifen treatment 4 weeks later (Figure 3E). Prior to tamoxifen administration, the percentages of donor-derived cells from $Dhx9^{f/f;ERT2}$ mice were comparable to those from $Dhx9^{f/f}$ controls (Figure S3F). However, after *Dhx9* depletion, a significant reduction in reconstitution was observed in PB of recipient mice (Figure S3G). At the end of 8 weeks, the contribution of $CD45.2^+$ donor-derived cells in total $CD45^+$, T ($CD3^+$), B ($B220^+$), and myeloid ($CD11b^+$) cells, and LSK, LK, and HSCs in the BM was drastically reduced in $Dhx9^{f/f;ERT2}$ transplant recipients (Figures S3H–S3K), confirming the defects associated with long-term HSC reconstitution. Collectively, these results suggest that the role of DHX9 in HSC maintenance *in vivo* is cell-intrinsic and is not due to defects in homing.

To further examine the effect of *Dhx9* loss on HSC self-renewal, we performed secondary BMT (Figure 3E). Notably, defective reconstitution of long-term hematopoiesis was demonstrated by decreased chimerism in PB for 16 weeks after secondary transplantation in $Dhx9^{f/f;ERT2}$ mice (Figures 3F and 3G). Similarly, reduced percentages of donor-derived total and multilineage cells, as well as HSPC populations, were observed in the BM of $Dhx9^{f/f;ERT2}$ mice at the endpoint of transplantation (Figures 3H–3J). These data indicate that *Dhx9*-deleted mice have inferior BM-repopulating LT-HSCs with reduced self-renewal. In summary, these data suggest that the loss of *Dhx9* impaired the long-term self-renewal capacity of HSCs.

DEAH-box helicase 9 sustains the hematopoietic gene expression program

To gain mechanistic insights into how DHX9 maintains HSC function, we characterized the transcriptome profiles of Lin^- cells in WT and cKO mice (Figure 4A). We identified 1321 significantly upregulated and 3235 downregulated genes after *Dhx9* knockout (fold change >2, $P_{adj} < 0.05$) (Figure 4B). Gene Ontology (GO) analysis of the upregulated genes showed enrichment in pathways associated with mitochondrion, cell cycle, and apoptosis in cKO cells (Figure 4C), which was consistent with increased ROS, cell cycle, and DNA damage observed in these cells (Figures 2E–2J). In addition, genes associated with hematopoiesis, HSC homeostasis, and cell differentiation were downregulated in cKO mice (Figure 4C). Consistent with the observed hematopoietic defects, gene set enrichment analysis (GSEA) revealed that the dramatically downregulated gene sets included those related to HSC quiescence (Forsberg et al., 2010) and LT-HSC signature (Figure 4D) (Ficara et al., 2008). Single-cell RNA-seq analysis of $Lin^-c\text{-}Kit^+$ cells further demonstrated that gene signatures associated with LT-HSC signature were markedly reduced in the HSC cluster of *Dhx9*-deficient mice, accompanied by a pronounced decrease in the proportion of HSCs and an increase in cycling HSC compared with controls (Figures S4A–S4C). Furthermore, motif analysis predicted the enrichment of PU.1 (SPI1), RUNX, and ERG DNA binding motifs in the promoters of these

All mice used in these experiments were analyzed seven days after the first poly(I:C) injection. The data are presented as the mean ± SEM. $*p < 0.05$; $**p < 0.01$; $***p < 0.001$; $****p < 0.0001$; ns, not statistically significant; unpaired two-tailed Student's *t* test.
See also Figure S2.

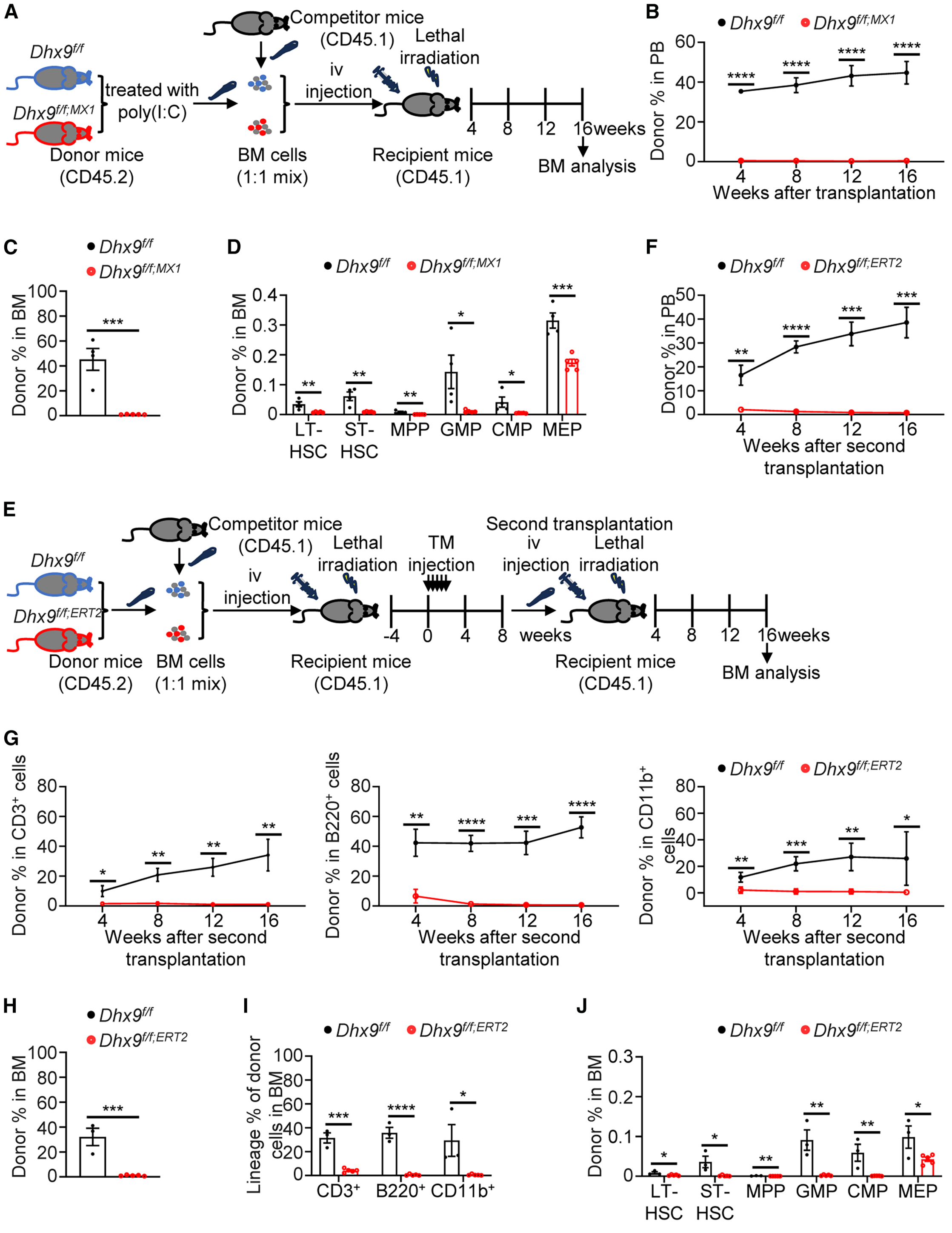

Figure 3. DHX9 is required for HSC reconstitution and self-renewal

(A) Schedule of competitive BMT. BM cells from *Dhx9*$^{f/f}$ or *Dhx9*$^{f/f;MX1}$ mice (CD45.2) were collected one week after the first dose of poly(I:C) injection and transplanted with WT competitor cells (CD45.1) into lethally irradiated WT recipients (CD45.1); iv, intravenous injection.
(B) CD45.2^{+} donor chimerism in the PB of recipient mice was measured every 4 weeks, and the results are graphed. WT ($n = 4$) and cKO ($n = 5$).
(C) Percentages of CD45.2^{+} cells in the BM of BMT recipient mice 16 weeks after transplantation. WT ($n = 4$) and cKO ($n = 5$).
(D) Percentages of CD45.2^{+} HSPC subpopulations in the BM of primary transplant recipient mice at 16 weeks. WT ($n = 4$) and cKO ($n = 5$).

(legend continued on next page)

downregulated genes (Figure 4E), which was consistent with BM failure. Among the significantly downregulated genes following *Dhx9* depletion, several key hematopoietic transcription factors that maintain HSC quiescence and self-renewal, including *Spi1*, *Cebpa*, *Gfi1*, *Xbp1*, *Fos*, *Hlf*, *Meis1,* and *Klf6,* as well as other critical stem cell genes such as *Mpl* and *Slfn2* were found (Figure 4F), which were further validated by qRT-PCR analysis (Figure 4G). In conclusion, these results indicate that DHX9 is required for the expression of hematopoiesis-maintenance genes.

DEAH-box helicase 9 interacts with CBP/p300 and maintains histone acetylation for transcriptional activation

Previous studies have shown that DHX9 binds to the transcriptional coactivators CBP/p300 and RNA Pol II at the promoter regions to regulate gene transcription (Nakajima et al., 1997; Ren et al., 2023). Based on this, we hypothesized that DHX9 cooperates with CBP/p300 in regulating HSC quiescence and self-renewal genes. We observed the colocalization of DHX9 and p300 in murine Lin^- cells via immunofluorescence (Figure 5A). This interaction was further confirmed through co-immunoprecipitation in 293T cells, where DHX9 was eluted from CBP immunoprecipitates (Figure 5B). Since CBP/p300 are well-known acetyltransferases (Weinert et al., 2018), we assessed histone acetylation and found that *Dhx9* depletion led to a global reduction in histone H3 acetylation, including the downregulation of histone H3 acetylation at lysines 9 and 27 (H3K9ac, H3K27ac) (Figures 5C–5F and S4D). We therefore hypothesized that DHX9 could sustain hematopoietic gene transcription in cooperation with histone acetylation by interacting with CBP/p300 at gene promoters.

To confirm our hypothesis, we additionally performed CUT&Tag analysis to characterize the genomic distribution of H3K27ac and DHX9 in murine Lin^- cells and compared the results with transcriptomic data to address their effects on hematopoietic gene transcription (Figure 4A). Peak analysis of DHX9-occupied regions revealed that DHX9 was localized mostly to distal intergenic (34.37%), other intron (23.72%), promoter (21.28%), and first intron (10.23%) regions (Figure S4E). Compared with random genomic distributions (Mould et al., 2015), these data suggest an enrichment of promoter localization of DHX9-binding regions. Furthermore, we found that genes whose promoter regions were bound by DHX9 were significantly downregulated in cKO Lin^- cells (Figure S4F), indicating a regulatory role of DHX9 in gene transcription. Motif analysis of DHX9-binding peaks revealed the enrichment of most hematopoietic transcription factors, such as PU.1, IRF8, and ERG DNA binding motifs (Figure S4G), which was consistent with the downregulated motifs in the transcriptomic results (Figure 4E).

Density map analysis of H3K27ac CUT&Tag signal revealed that H3K27ac in all gene transcription start sites (TSS) ± 10 kb regions was moderately decreased in cKO cells (Figure 5G). Specifically, we found that loss of H3K27ac occupancy in TSS regions was strongly associated with transcriptional downregulation upon DHX9 depletion (Figure 5H). In addition, hematopoietic cell lineage signatures and related TF motifs, such as PU.1, which was among the decreased H3K27ac motifs, as well as the DHX9-binding motif (Figures 5I, S4G, and S4H), were consistent with the transcriptional profiles following *Dhx9* deletion (Figure 4).

To further investigate the role of H3K27ac in DHX9-mediated transcriptional regulation of HSC function, we assessed H3K27ac enrichment at the promoter regions of key hematopoietic genes that are significantly downregulated upon *Dhx9* deletion. Analysis using the IGV genome browser revealed a reduction in H3K27ac peaks at these hematopoietic genes in cKO cells compared to controls. Notably, these genes also coincided with DHX9-binding sites (Figures 5J and S4I). The decreased H3K27ac levels and DHX9 occupancy across these loci were validated by chromatin immunoprecipitation (ChIP)-qPCR (Figures 5K, S4J, and S4K). Electrophoretic mobility shift assays (EMSA) were performed using biotin-labeled DNA probes derived from the XBP1 promoter region identified by CUT&Tag. Incubation with nuclear extracts from DHX9-overexpressing 293T cells led to a supershift upon the addition of a DHX9 antibody (Figure S4L), suggesting that DHX9 functionally associates with chromatin and may promote CBP/p300-mediated histone acetylation. To further confirm the involvement of H3K27ac in DHX9-driven gene expression, we treated $Dhx9^{f/f}$ -hematopoietic cells with the CBP/p300

(E) Schematic of competitive reconstitution assays using the UBC-CreERT2 system. BM cells from 8-week-old $Dhx9^{f/f}$ and $Dhx9^{f/f;ERT2}$ mice (CD45.2) were transplanted into lethally irradiated WT recipient mice (CD45.1) with the same number of competitor BM cells (CD45.1) (5×10^5 cells). Four weeks after transplantation, the recipient mice were injected with tamoxifen 5 times every second day. PB analyses were performed every 4 weeks, and BM analysis was performed 8 weeks after tamoxifen injection, while secondary BMT was performed by injecting 1×10^6 total BM cells from mice receiving primary transplants into secondary recipients. TM, tamoxifen.
(F–J) The contribution of $CD45.2^+$ cells in total $CD45^+$, T, B, and myeloid cells from PB (F, G) or BM (H, I), and HSCs and other hematopoietic populations in the BM (J) was analyzed in secondary BMT recipients. $Dhx9^{f/f}$ ($n = 3$) and $Dhx9^{f/f;ERT2}$ ($n = 5$).
The data are presented as the mean ± SEM. *$p < 0.05$; **$p < 0.01$; ***$p < 0.001$; and ****$p < 0.0001$; unpaired two-tailed Student's t test. See also Figure S3.

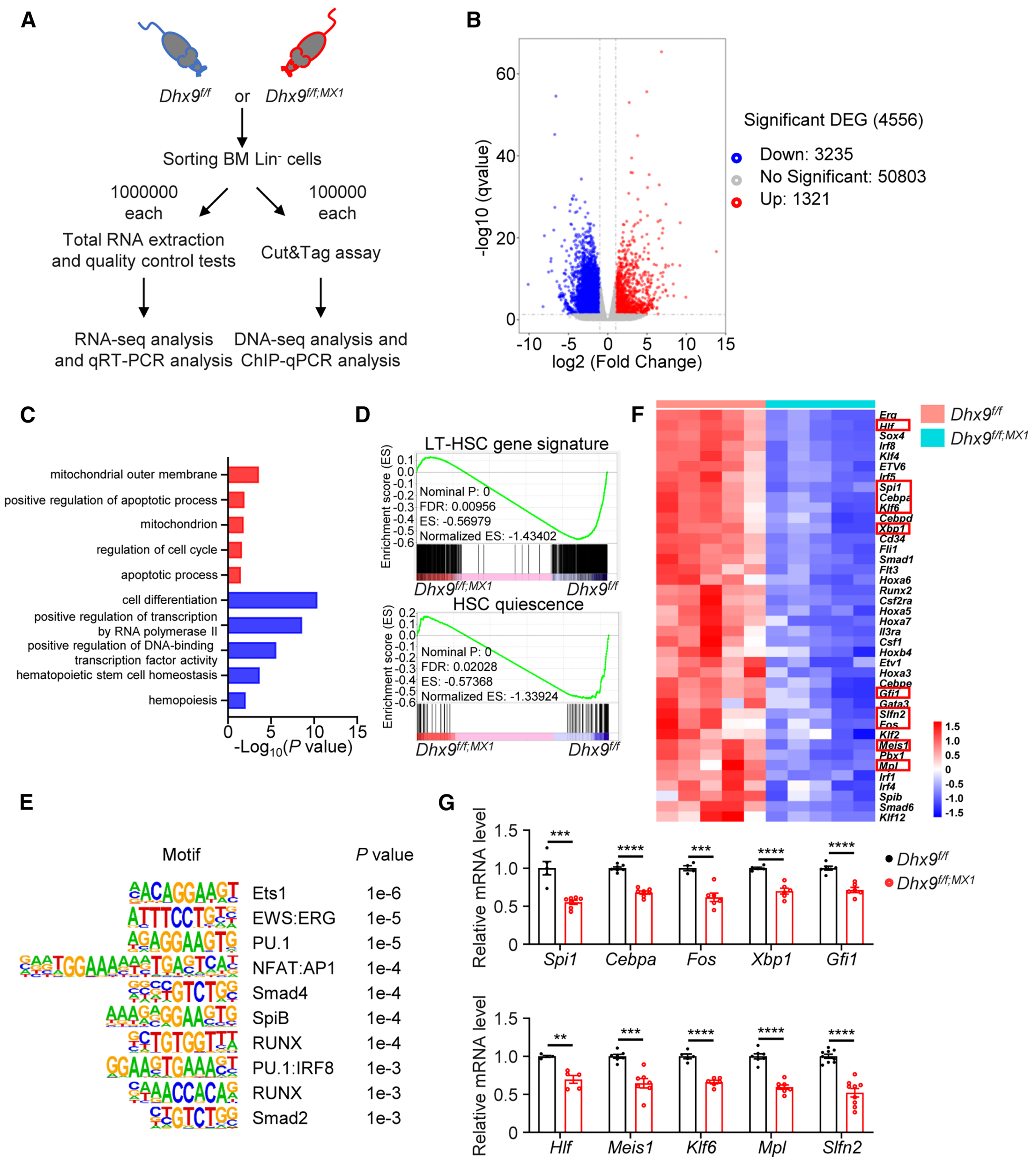

Figure 4. DHX9 sustains the hematopoietic gene expression program

(A) Schematic illustration of the experimental design.

(B) Volcano plot shows the genes differentially expressed between WT and cKO Lin⁻ hematopoietic cells. Genes with an adjusted *p* value (P_{adj}) < 0.05 are highlighted (blue: genes downregulated in cKO Lin⁻ cells; red: genes upregulated in cKO Lin⁻ cells).

(C) Pathway analysis of genes associated with RNA-seq downregulated (blue) or upregulated (red) regions in cKO Lin⁻ cells.

(D) GSEA of cKO Lin⁻ hematopoietic cells compared with WT Lin⁻ hematopoietic cells. ES, enrichment score; FDR, false discovery rate.

(E) Transcription factor motifs enriched in TSS (−1 kb to +1 bp) of cKO Lin⁻ cell-downregulated genes.

(F) Heatmap shows hematopoiesis-related genes, as revealed by transcriptomic studies. The genes in red boxes represent critical hematopoietic genes for maintaining HSC quiescence and self-renewal.

(G) mRNA expression of the significantly changed hematopoiesis-related genes in cKO Lin⁻ hematopoietic cells ($n \geq 4$).

The data are presented as the mean ± SEM. $**p < 0.01$; $***p < 0.001$; $****p < 0.0001$; unpaired two-tailed Student's *t* test.

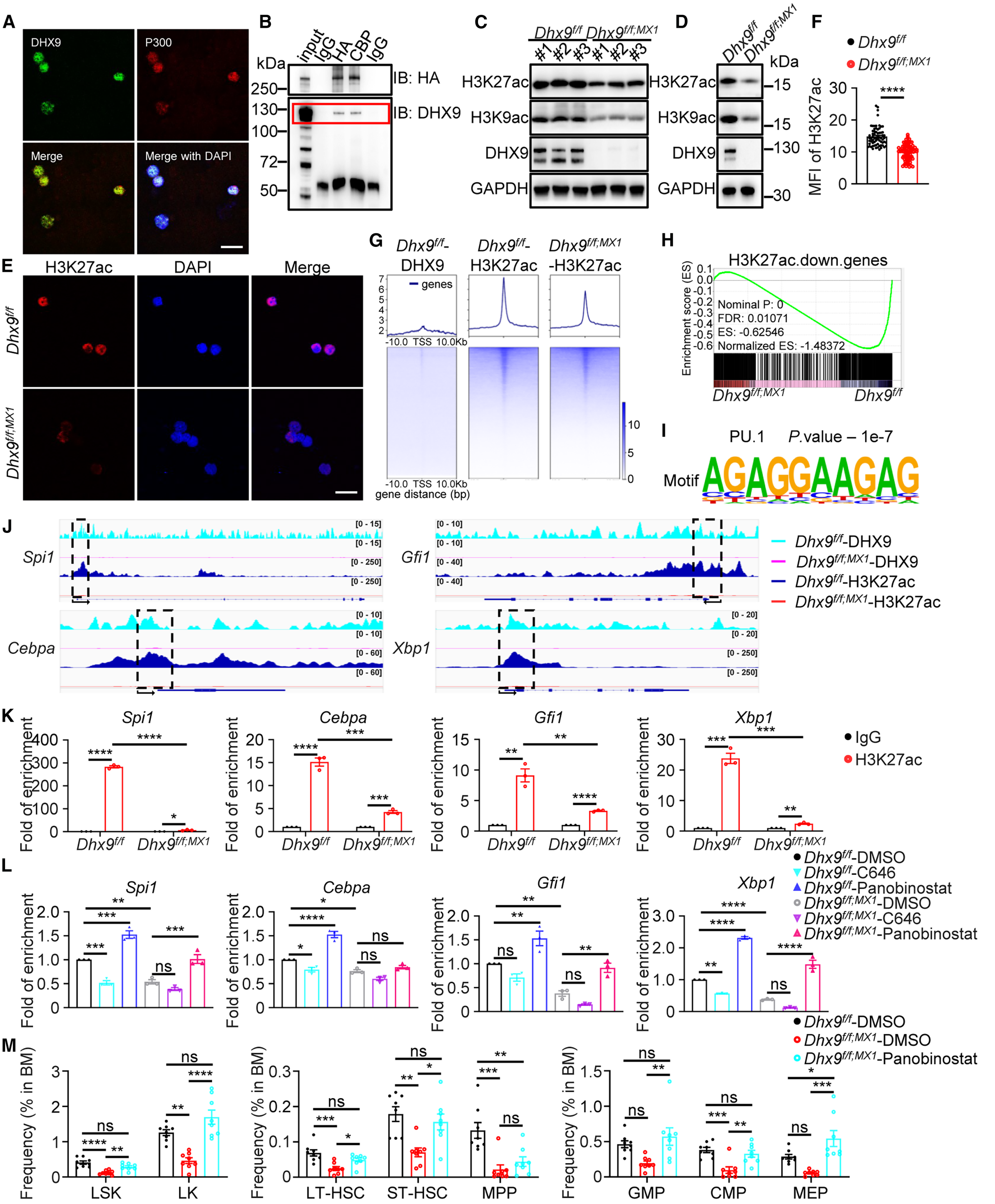

(legend on next page)

inhibitor C646, resulting in a downregulation of key hematopoietic genes. Conversely, exposure to the broad-spectrum histone deacetylase inhibitor (HDACi) panobinostat increased the expression of these genes in WT cells. Similar expression trends were observed in cKO cells, although the magnitude of these changes was generally less pronounced (Figures 5L, S5A, and S5B).

To further explore the role of H3K27ac in hematopoiesis *in vivo*, cKO mice were treated with panobinostat. Panobinostat treatment markedly increased the ratios of multiple HSPC populations, with a trend toward higher total BM cellularity (Figures 5M, S5C, and S5D). Collectively, these data suggest that DHX9 cooperates with CBP/p300 to preserve H3K27 acetylation, thereby maintaining hematopoietic gene expression.

Restoring H3K27ac levels partially rescues hematopoietic defects caused by DEAH-box helicase 9 inhibition in umbilical cord blood hematopoietic stem cells

We next investigated whether the functional cooperation of DHX9 and H3K27ac could be recapitulated in human HSPCs. $CD34^+$ cells were sorted from human umbilical cord blood (UCB) cells and were treated with ATX968, a DHX9 inhibitor (Castro et al., 2025) for 6 consecutive days. We observed that ATX968 significantly reduced total cell numbers in $CD34^+$ cells compared to controls (Figure 6A). Flow cytometric analysis revealed that DHX9 inhibition led to marked reductions in $CD34^+CD38^-$, $CD34^+CD38^+$, LMPP ($CD34^+CD38^-CD45RA^+CD90^-$), MPP ($CD34^+CD38^-CD45RA^-CD90^-$), GMP ($CD34^+CD38^+CD45RA^+$), CMP ($CD34^+CD38^{high}CD45RA^-$), and MEP ($CD34^+CD38^{low}CD45RA^-$) (Figures 6B and S6A–S6C). CFU assays further showed that ATX968 treatment significantly reduced the colony numbers of CFU-GEMM, CFU-GM, BFU-E, and erythroid colony-forming units (CFU-E) (Figures 6C and 6D). This data suggests that DHX9 inhibition impaired hematopoiesis was functionally relevant but not simply a change in cell surface marker expression. In line with *in vivo* data in *Dhx9*-deficient mice (Figures 2E and 2F), ATX968 treatment significantly increased apoptosis compared to controls in $CD34^+$ cells (Figures 6E and 6F), supporting a role of DHX9 in maintaining HSPC survival.

To test whether restoring histone acetylation could rescue the impaired hematopoietic phenotype, we co-treated $CD34^+$ cells with ATX968 and panobinostat. Strikingly, panobinostat co-treatment significantly restored HSPC numbers and reduced apoptosis (Figures 6G, 6H, and S6D–S6F). These findings suggest that DHX9 is essential for the maintenance of human hematopoietic homeostasis, at least in part through its regulation of H3K27 acetylation. Notably, the capacity of HDACi to reverse ATX968-induced defects underscores its therapeutic potential for mitigating hematopoietic dysfunction caused by DHX9 impairment.

DISCUSSION

The mechanisms underlying self-renewal and multipotent capabilities of HSCs remain incompletely understood (Wilkinson et al., 2020). We found that DHX9 is required

Figure 5. Cooperation between DHX9 and CBP/p300 maintains hematopoietic homeostasis via H3K27 acetylation

(A) Representative confocal microscopy images of $Dhx9^{f/f}$ Lin^- cells staining by DHX9 and p300 are shown as indicated. Scale bars, 20 μm.
(B) 293T cells were co-transfected with pLVX-*DHX9* and pEnCMV-*CREBBP*-HA-SV40-Neo. After 48 h, cell lysates were collected, then immunoprecipitated, and blotted as indicated.
(C and D) Western blot analyses of H3K9ac and H3K27ac in TNCs (C) or freshly isolated Lin^- cells (D) from the BM of WT and cKO mice.
(E and F) Confocal microscopy images (E) and quantification of H3K27ac MFI (F) in WT and cKO Lin^- cells. Each dot represents a single cell from 3 mice per group. Scale bars, 20 μm.
(G) Heatmaps show CUT&Tag signals of DHX9 or H3K27ac centered on the TSS region in Lin^- cells sorted from WT and cKO mice.
(H) Leading-edge plots show the enrichment of genes associated with H3K27ac-downregulated regions based on GSEA of WT and cKO Lin^- cells. ES, enrichment score; FDR, false discovery rate.
(I) Transcription factor motifs enriched in H3K27ac genomic regions downregulated in cKO Lin^- cells.
(J) IGV genome browser tracks show CUT&Tag sequencing data of DHX9 (WT Lin^- cells)–, DHX9 (cKO Lin^- cells)–, H3K27ac (WT Lin^- cells)–, and H3K27ac (cKO Lin^- cells)–bound promoter regions of hematopoiesis-related genes.
(K) ChIP-qPCR analysis of H3K27ac-bound sites in hematopoiesis-related gene (*Spi1*, *Cebpa*, *Gfi1*, *Xbp1*) promoters with or without *Dhx9* ($n = 3$).
(L) qRT-PCR analysis of hematopoiesis-related genes in WT and cKO hematopoietic cells after treatment with 40 μM C646 or 20 nM panobinostat ($n = 3$).
(M) Ratios of different HSPC populations in the BM of mice treated with DMSO or 5 mg/kg panobinostat via intraperitoneal injection every two days for a total of five doses, starting seven days after the first poly(I:C) injection ($n = 8$).
The data are presented as the mean ± SEM. $*p < 0.05$; $**p < 0.01$; $***p < 0.001$; $****p < 0.0001$; ns, not statistically significant; unpaired two-tailed Student's t test (F and K), one-way ANOVA with Tukey's test (L and M).
See also Figures S4 and S5.

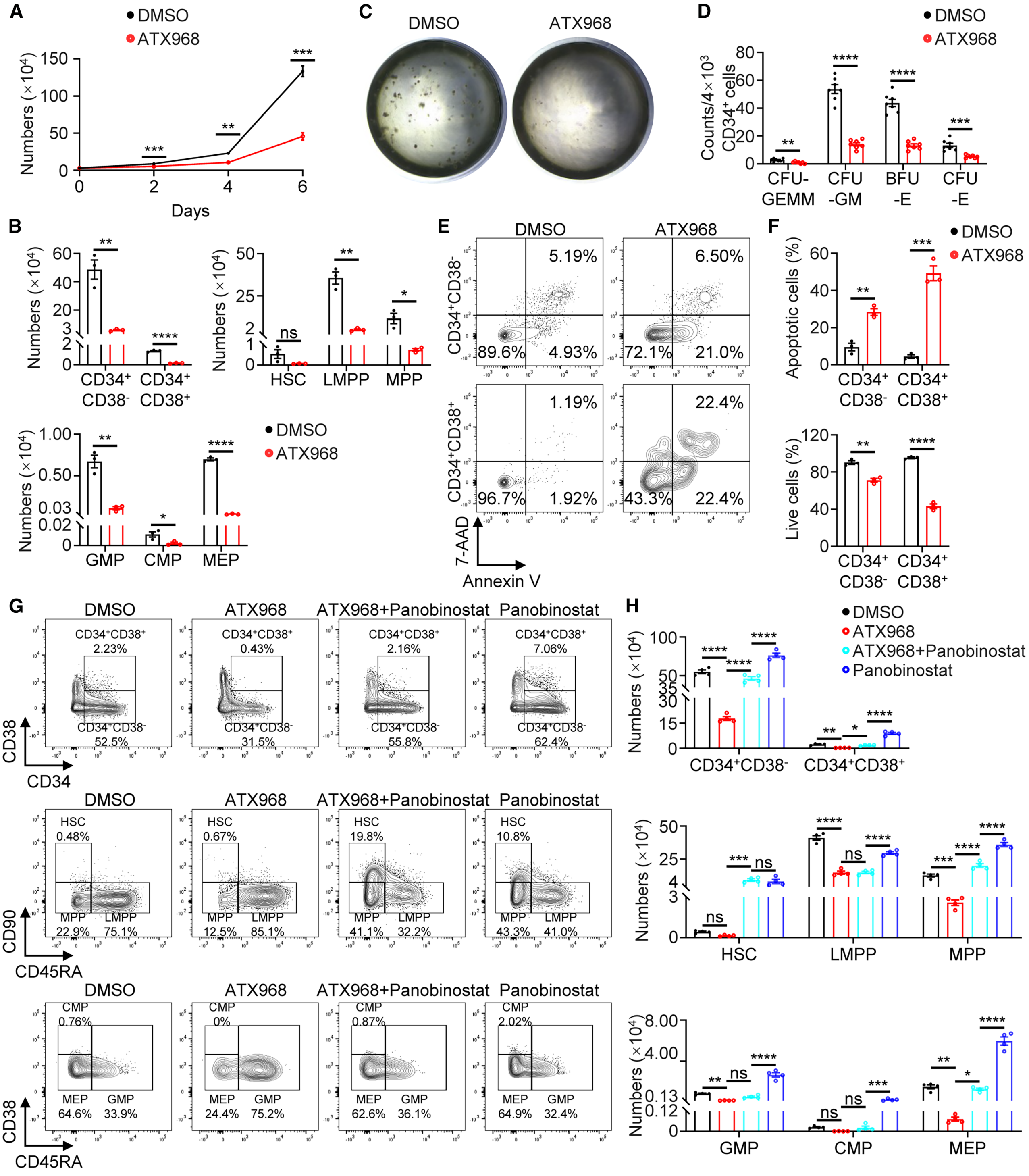

Figure 6. Restoring H3K27ac levels partially rescues the damage caused by DHX9 inhibition in umbilical cord blood HSCs

(A) Growth curves of CD34$^+$ cells following treatment with DMSO or 40 μM ATX968 (*n* = 3).

(B) Absolute numbers of different HSPC populations analyzed by flow cytometry following 6-day treatment with DMSO or ATX968 treatment (*n* = 3).

(C) Representative images of the colony-forming unit assay.

(D) Hematopoietic colonies were enumerated (*n* = 7).

(legend continued on next page)

for multilineage hematopoiesis under steady-state conditions. Deletion of *Dhx9* results in decreased cellularity in the spleen, thymus, and BM, as well as decreased WBCs and RBCs in the PB. Given that global *Dhx9* knockout is embryonic lethal (Lee et al., 1998), its effects on hematopoiesis could potentially reflect broad impacts on cell survival rather than selective hematopoietic defects. To address this, we performed competitive BMT, which revealed that *Dhx9*-deficient cells failed to reconstitute hematopoiesis in primary and secondary recipients (Figure 3), suggesting a critical role of DHX9 in HSC self-renewal. Additionally, apoptosis and cellular stress assessment further demonstrated that DHX9 maintains HSC mainly by sustaining its survival and self-renewal capacity beyond general effects on cell viability (Figures 2, 4, and 5). It is worth noting that our transplantation experiments were performed using total BM cells rather than highly purified HSCs, and genotype-dependent variation in HSPC abundance may have partially contributed to the observed reconstitution outcomes. We also cannot completely rule out the possibility that rare HSC clones escaping DHX9 deletion disproportionately contributed to donor chimerism. Therefore, these data should be interpreted with caution. Future purified HSC transplantation studies are needed to clarify the role of DHX9 in sustaining HSCs, and analyses of donor-derived cells could further validate this conclusion. A similar consideration applies to the RNA-seq and CUT&Tag experiments performed on Lin^- cells, where differences in HSPC composition between genotypes could have partially influenced the resulting transcriptional and chromatin landscapes.

Interestingly, in *Dhx9*$^{f/f;Vav1}$ mice, although HSC and multiple progenitor cell types were markedly reduced (Figure 1H), PLT counts remained unchanged (Figure S1H). This likely reflects the strong buffering capacity of PLT homeostasis, which is regulated not only by the progenitor pool but also by megakaryocyte maturation, ploidy, shear stress-mediated PLT release, and microenvironmental cues (Furniss et al., 2024). In addition, DHX9 loss may reshape megakaryopoiesis. The remaining *Dhx9*-deficient MEPs and megakaryocytes could become functionally hyperactive, exhibiting higher ploidy and enhanced PLT formation. Alternatively, *Dhx9* deficiency may induce a chronic low-grade stress state that subtly biases HSCs (Haas et al., 2015; Roch et al., 2015) or primes MEPs toward increased megakaryocytic differentiation, resembling sustained "emergency" PLT production. Consistent with these possibilities, our flow cytometric (Figure S2K) and single-cell RNA-seq analyses (Figure S4B) suggest that DHX9 loss preferentially impairs the erythroid differentiation potential of MEPs while largely preserving megakaryocytic differentiation.

DHX9 is a multifunctional helicase that acts on both RNA and DNA substrates, including complex nucleic acid structures such as R-loops, G-quadruplexes, and H-DNA (Liu et al., 2024b). DHX9 participates in diverse roles involving RNA processing, gene transcription, and translation. Previous studies have reported that DHX9 can bind to transcription factors and activate gene transcription in various cell contexts (Huo et al., 2010; xRen et al., 2023). Here, we found transcriptional profiling of *Dhx9*-deficient cells showed a significant downregulation of hematopoietic genes, including *Spi1*, *Cebpa*, *Gfi1*, and *Xbp1*, which are essential for maintaining HSC quiescence and self-renewal. Mechanistically, we found that DHX9 could colocalize with CBP/p300 and promote H3 acetylation. Consistent with prior reports highlighting the role of H3K27ac in sustaining HSC self-renewal and lineage priming (Chagraoui et al., 2021; Liu et al., 2024a), we observed that H3K27ac binding peaks were decreased in *Dhx9*$^{f/f;MX1}$ cells on TSS regions of the whole genome, especially on promoters of key hematopoietic regulators. These findings suggest that DHX9 functions as a coactivator or chromatin scaffold that facilitates the recruitment or stabilization of CBP/p300 at promoter regions of hematopoietic regulatory genes to maintain high levels of H3K27 acetylation and robust transcriptional activity. In line with this, pharmacological inhibition of CBP/p300 by C646 mimicked the transcriptional defects observed in *Dhx9*-deficient cells, whereas the restoration of histone acetylation with the broad-spectrum HDACi panobinostat partially rescued these impairments. Although these results imply that DHX9 contributes to transcriptional activation by cooperating with CBP/p300, whether DHX9 binds DNA directly or acts indirectly through associated protein complexes remains to be determined. As a full-length purified DHX9 protein is currently unavailable, future experiments using purified DHX9 and *in vitro* DNA binding assays will be required to resolve this question.

(E and F) Representative gating strategies used in FACS analyses for apoptotic cells (E) and quantification of apoptosis ratios (F) ($n = 3$).
(G) Representative gating strategies used in FACS analyses for the frequencies of $CD34^+CD38^-$, $CD34^+CD38^+$ cells, and indicated lineage progenitors.
(H) Absolute numbers of different HSPC populations analyzed by flow cytometry following 6-day treatment with DMSO, ATX968, and/or 1 nM panobinostat treatment ($n = 4$).
The data are presented as the mean ± SEM. *$p < 0.05$; **$p < 0.01$; ***$p < 0.001$; ****$p < 0.0001$; ns, not statistically significant; unpaired two-tailed Student's t test (A, B, D, and F), one-way ANOVA with Tukey's test (H).
See also Figure S6.

To further test this relationship, we treated *Dhx9*-deficient mice or ATX968-treated human UCB cells with panobinostat. Interestingly, panobinostat restored the frequencies of LT-HSCs, ST-HSCs, CMPs, GMPs, and MEPs in *Dhx9*-deficient mice, but failed to rescue MPPs. In contrast, panobinostat effectively rescued MPP defects caused by DHX9 inhibition in human UCB HSCs. This discrepancy may reflect distinct regulatory dependencies between murine and human hematopoiesis. Indeed, recent single-cell analyses have revealed that while the overall hematopoietic hierarchy is conserved between mice and humans, subtle differences exist in specific hematopoietic populations and regulatory programs (Buenrostro et al., 2018; Gao et al., 2021; Kucinski et al., 2024). Additionally, complete loss of DHX9 in mice may induce irreversible transcriptional or epigenetic alterations in MPPs that cannot be fully compensated by HDACi, whereas transient DHX9 inhibition in human HSCs allows panobinostat to restore both MPP phenotype and function.

It should be noted that panobinostat is a non-selective HDACi and not specific to H3K27ac. Our analysis focused on H3K27ac as a representative active histone mark given its established role in HSC regulation, but panobinostat likely induces broad chromatin changes, including increases in other active acetylation marks (H3K9ac, H4ac) and decreases in repressive marks (H3K9me3) (Legoff et al., 2021; Moshref et al., 2021). Therefore, while our data indicate that elevated H3K27ac correlates with rescue of the DHX9-deficient phenotype, we cannot fully exclude potential indirect effects arising from these broader epigenetic alterations.

Dhx9 deficiency caused a decrease in H3K27ac and downregulated the expression of hematopoietic regulators. Since the deregulation of histone marks in HSCs could predetermine stem cell aging and the development of myeloid malignancies (Adelman et al., 2019; Verovskaya et al., 2019), we speculate that DHX9 could play a role in myeloid malignancies. Indeed, a recent study has suggested an oncogenic role for DHX9 in acute myeloid leukemia (AML) (Xiong et al., 2025). Notably, the therapeutic implications of targeting DHX9 may differ fundamentally depending on disease context. In hematologic malignancies such as AML, where DHX9 may be aberrantly activated or co-opted by oncogenic programs, inhibiting its helicase activity could suppress leukemogenic chromatin remodeling and gene expression. In contrast, in BM failure syndromes characterized by reduced DHX9 expression or function, our findings raise the possibility that restoring downstream chromatin acetylation—such as through HDACi—may help rescue impaired gene expression and hematopoietic output. These divergent roles underscore the need for disease-specific strategies when targeting the DHX9–CBP/p300–H3K27ac regulatory axis and highlight the broader principle that chromatin-modifying factors may act as context-dependent therapeutic vulnerabilities in hematopoietic disorders.

In summary, we identify DHX9 as a critical regulator of hematopoietic gene transcription and HSC function. Our findings underscore the importance of a permissive chromatin landscape in maintaining HSC quiescence and self-renewal, offering mechanistic insights and potential therapeutic avenues for diseases associated with the epigenetic dysregulation of hematopoiesis.

METHODS

Animals

For tissue-specific or inducible deletion of *Dhx9*, *Dhx9*$^{f/f}$ mice were crossed with Vav1-Cre, MX1-Cre, or UBC-CreERT2 (The Jackson Laboratory; JAX:007179) mice. MX1-Cre mice were injected intraperitoneally with 250 μg poly (I:C) every other day for 3 doses. Mice were analyzed 7 days after the first poly(I:C) administration unless otherwise indicated. For the induction of UBC-CreERT2 activity, the mice (aged 6–8 weeks) were injected intraperitoneally with five doses of tamoxifen dissolved in corn oil, 120 mg/kg body weight per dose, doses separated by 48 h. Mouse sex was randomized. The mice were maintained in a specific pathogen-free facility under strict 12:12 h light−dark cycles, with lights on from 8 a.m. to 8 p.m. All animal procedures were approved by the Ethics Committee of USTC (USTCACUC26120123058).

Competitive repopulation assay

For competitive BMT, 5×10^5 total BM cells from *Dhx9*$^{f/f}$ or *Dhx9*$^{f/f;MX1}$ (CD45.2) or *Dhx9*$^{f/f;ERT2}$ (CD45.2) mice were mixed with the same number of competitor total BM cells (CD45.1), and transplanted into lethally irradiated (a split dose of 10.5 Gy) recipient mice (CD45.1) via intravenous injection. Reconstituted donor HSPCs from the BM were analyzed after 16 weeks of BMT, and 1×10^6 BM cells obtained from primary recipient mice were transplanted into lethally irradiated (10.5 Gy) secondary WT (CD45.1) recipient mice for the second transplantation.

Flow cytometry

For mouse HSPCs staining, BM cells were harvested, treated with ACK lysis buffer, and incubated with Alexa Fluor 700 anti-mouse CD34 at 4°C for 1 h, further incubated with a cocktail of PE-conjugated primary antibodies containing mouse specific lineage markers: anti-CD2, anti-Ter119, anti-CD3ε, anti-Gr-1, anti-B220 and anti-CD41, Pacific Blue anti-mouse Sca-1, APC anti-mouse c-Kit, Brilliant Violet 510 anti-mouse CD45, PE-CF594 Rat anti-mouse Flk2, and FITC anti-mouse CD16/32, or FITC anti-mouse CD48, PE/Cy7 anti-mouse CD150 at 4°C for 0.5 h. For

the analysis of megakaryocyte progenitors (MkPs: $CD45^{+}Lin^{-}Sca\text{-}1^{-}c\text{-}Kit^{+}CD41^{+}CD150^{+}$), BM cells were stained with antibodies against CD45, c-Kit, Sca-1, CD150, CD48, CD41 and Lineage Cocktail. For the lineage cell analyses, cells from BM and PB were stained with antibodies against CD45.1, CD45.2, B220, CD3, and CD11b. For the reconstituted HSPCs, BM cells were stained with the lineage cocktail and antibodies against Sca-1, c-Kit, CD16/CD32, CD34, Flk2, CD45.1, and CD45.2.

For human HSPCs staining, cultured cells were collected on the sixth day after treatment, washed once with PBS, and incubated with antibodies against lineage cocktail, CD34, CD90, CD38, and CD45RA at 4°C for 30 min. Flow cytometry was performed on a BD LSR FORTESSA, and data were analyzed using FlowJo V10.

RNA sequencing and analysis

Lin^{-} cells were sorted from the BM of *Dhx9*$^{f/f}$ and *Dhx9*$^{f/f;MX1}$ mice. Total RNA was isolated via the use of TRIzol reagent. Libraries were prepared and sequenced on the Illumina NovaSeq 6000 platform with a 2×150 paired-end configuration. The raw data were processed by filtering out reads containing adapters, PCR primers, or fragments thereof, and reads whose quality was lower than 20, to obtain clean read data, which were used for all subsequent analyses. The clean data were initially aligned to the mouse genome (GRCm39) via HISAT2 software (v2.0.1). Differential gene expression analyses were conducted using the DESeq2 Bioconductor package. Genes with an adjusted p value <0.05 and an absolute fold change (FC) $\geq$ 2 were considered significantly differentially expressed genes.

Quantification and statistical analysis

All statistical analyses were performed by GraphPad Prism 8. The error bars represent the standard error of the mean (SEM). p values were calculated by two-tailed unpaired t tests for comparisons between two groups, by one-way ANOVA with Dunnett's or Tukey's test for comparisons among more than two groups. $p < 0.05$ (confidence interval of 95%) was considered statistically significant. In the Figures, *, **, ***, and **** were used to indicate $p < 0.05$, 0.01, 0.001, and 0.0001, respectively. Statistical parameters were indicated in the figure legends. In our study, n refers to the number of biological replicates from individual mice or UCB samples analyzed per group, unless otherwise stated. For all other experiments, $n \geq 3$, and the experiments were performed in three or more fully independent replicates.

RESOURCE AVAILABILITY

Lead contact

Further information and requests for resources and reagents should be directed to and will be fulfilled by the lead contact, Huaiping Zhu (huaipingzhu@ustc.edu.cn).

Materials availability

This study did not generate new unique reagents.

Data and code availability

- RNA-seq and CUT&Tag sequencing data have been deposited at Gene Expression Omnibus (GEO: GSE278362 and GSE278361), and scRNA-seq data have been deposited at Sequence Read Archive (SRA: PRJNA1356327 ; https://www.ncbi.nlm.nih.gov/sra/PRJNA1356327). All datasets are publicly available as of the date of publication.
- Original Western blot images have been deposited at Mendeley Data at [Reserved https://doi.org/10.17632/wvpdmpx7v5.1] and are publicly available as of the date of publication. Microscopy data reported in this article will be shared by the lead contact upon request.
- This article does not report original code.
- Any additional information required to reanalyze the data reported in this article is available from the lead contact upon request.

ACKNOWLEDGMENTS

We thank Zhongjun Dong for gifting the Vav1-Cre mice and Yongzhong Liu for gifting the MX1-Cre mice. We thank Lijun Xia for insightful discussions. This work was supported by grants from the National Natural Science Foundation of China [(31870897) (H.Z.) and (82200197) (M.G)].

AUTHOR CONTRIBUTIONS

M.S. and M.G. designed and performed the experiments, analyzed the data, and wrote the article; H.L., C.W., X.H., Y.X., and Y.C. helped to conduct the animal experiments; S.Z. and X.R. provided scientific counseling and materials; H.Z. conceived the project, designed the study, and revised the article; all authors discussed the article.

DECLARATION OF INTERESTS

The authors declare no competing interests.

DECLARATION OF GENERATIVE AI AND AI-ASSISTED TECHNOLOGIES IN THE WRITING PROCESS

The authors declare that no generative AI or AI-assisted technologies were used in the preparation of this work.

SUPPLEMENTAL INFORMATION

Supplemental information can be found online at https://doi.org/10.1016/j.stemcr.2026.102794.

Received: June 23, 2025
Revised: January 6, 2026
Accepted: January 7, 2026
Published: February 5, 2026

REFERENCES

Adelman, E.R., Huang, H.T., Roisman, A., Olsson, A., Colaprico, A., Qin, T., Lindsley, R.C., Bejar, R., Salomonis, N., Grimes, H.L., and

Figueroa, M.E. (2019). Aging Human Hematopoietic Stem Cells Manifest Profound Epigenetic Reprogramming of Enhancers That May Predispose to Leukemia. Cancer Discov. *9*, 1080–1101. https://doi.org/10.1158/2159-8290.Cd-18-1474.

Boulais, P.E., and Frenette, P.S. (2015). Making sense of hematopoietic stem cell niches. Blood *125*, 2621–2629. https://doi.org/10.1182/blood-2014-09-570192.

Buenrostro, J.D., Corces, M.R., Lareau, C.A., Wu, B., Schep, A.N., Aryee, M.J., Majeti, R., Chang, H.Y., and Greenleaf, W.J. (2018). Integrated Single-Cell Analysis Maps the Continuous Regulatory Landscape of Human Hematopoietic Differentiation. Cell *173*, 1535–1548.e16. https://doi.org/10.1016/j.cell.2018.03.074.

Butler, J.S., and Dent, S.Y.R. (2013). The role of chromatin modifiers in normal and malignant hematopoiesis. Blood *121*, 3076–3084. https://doi.org/10.1182/blood-2012-10-451237.

Castro, J., Daniels, M.H., Brennan, D., Johnston, B., Gotur, D., Lee, Y.T., Knockenhauer, K.E., Lu, C., Wu, J., Nayak, S., et al. (2025). A Potent, Selective, Small-Molecule Inhibitor of DHX9 Abrogates Proliferation of Microsatellite Instable Cancers with Deficient Mismatch Repair. Cancer Res. *85*, 758–776. https://doi.org/10.1158/0008-5472.Can-24-0397.

Chagraoui, J., Girard, S., Spinella, J.F., Simon, L., Bonneil, E., Mayotte, N., MacRae, T., Coulombe-Huntington, J., Bertomeu, T., Moison, C., et al. (2021). UM171 Preserves Epigenetic Marks that Are Reduced in Ex Vivo Culture of Human HSCs via Potentiation of the CLR3-KBTBD4 Complex. Cell Stem Cell *28*, 48–62.e6. https://doi.org/10.1016/j.stem.2020.12.002.

Dzierzak, E., and Bigas, A. (2018). Blood Development: Hematopoietic Stem Cell Dependence and Independence. Cell Stem Cell *22*, 639–651. https://doi.org/10.1016/j.stem.2018.04.015.

Ficara, F., Murphy, M.J., Lin, M., and Cleary, M.L. (2008). Pbx1 regulates self-renewal of long-term hematopoietic stem cells by maintaining their quiescence. Cell Stem Cell *2*, 484–496. https://doi.org/10.1016/j.stem.2008.03.004.

Forsberg, E.C., Passegué, E., Prohaska, S.S., Wagers, A.J., Koeva, M., Stuart, J.M., and Weissman, I.L. (2010). Molecular signatures of quiescent, mobilized and leukemia-initiating hematopoietic stem cells. PLoS One *5*, e8785. https://doi.org/10.1371/journal.pone.0008785.

Furniss, J.A., Tarassova, N., and Poole, A.W. (2024). Platelet generation in vivo and in vitro. Blood *144*, 2283–2294. https://doi.org/10.1182/blood.2024024601.

Gao, S., Wu, Z., Kannan, J., Mathews, L., Feng, X., Kajigaya, S., and Young, N.S. (2021). Comparative Transcriptomic Analysis of the Hematopoietic System between Human and Mouse by Single Cell RNA Sequencing. Cells *10*, 973. https://doi.org/10.3390/cells10050973.

Haas, S., Hansson, J., Klimmeck, D., Loeffler, D., Velten, L., Uckelmann, H., Wurzer, S., Prendergast, Á.M., Schnell, A., Hexel, K., et al. (2015). Inflammation-Induced Emergency Megakaryopoiesis Driven by Hematopoietic Stem Cell-like Megakaryocyte Progenitors. Cell Stem Cell *17*, 422–434. https://doi.org/10.1016/j.stem.2015.07.007.

Hu, M., Zeng, H., Chen, S., Xu, Y., Wang, S., Tang, Y., Wang, X., Du, C., Shen, M., Chen, F., et al. (2018). SRC-3 is involved in maintaining hematopoietic stem cell quiescence by regulation of mitochondrial metabolism in mice. Blood *132*, 911–923. https://doi.org/10.1182/blood-2018-02-831669.

Huo, L., Wang, Y.N., Xia, W., Hsu, S.C., Lai, C.C., Li, L.Y., Chang, W.C., Wang, Y., Hsu, M.C., Yu, Y.L., et al. (2010). RNA helicase A is a DNA-binding partner for EGFR-mediated transcriptional activation in the nucleus. Proc. Natl. Acad. Sci. USA *107*, 16125–16130. https://doi.org/10.1073/pnas.1000743107.

Kucinski, I., Campos, J., Barile, M., Severi, F., Bohin, N., Moreira, P.N., Allen, L., Lawson, H., Haltalli, M.L.R., Kinston, S.J., et al. (2024). A time- and single-cell-resolved model of murine bone marrow hematopoiesis. Cell Stem Cell *31*, 244–259.e10. https://doi.org/10.1016/j.stem.2023.12.001.

Lee, C.G., da Costa Soares, V., Newberger, C., Manova, K., Lacy, E., and Hurwitz, J. (1998). RNA helicase A is essential for normal gastrulation. Proc. Natl. Acad. Sci. USA *95*, 13709–13713. https://doi.org/10.1073/pnas.95.23.13709.

Legoff, L., Dali, O., De La Mata Santaella, E., Jaulin, C., D'Cruz, S.C., and Smagulova, F. (2021). Histone deacetylase inhibition leads to regulatory histone mark alterations and impairs meiosis in oocytes. Epigenetics Chromatin *14*, 39. https://doi.org/10.1186/s13072-021-00413-8.

Leone, S., Bär, D., Slabber, C.F., Dalcher, D., and Santoro, R. (2017). The RNA helicase DHX9 establishes nucleolar heterochromatin, and this activity is required for embryonic stem cell differentiation. EMBO Rep. *18*, 1248–1262. https://doi.org/10.15252/embr.201744330.

Liu, G., Kim, J., Nguyen, N., Zhou, L., and Dean, A. (2024a). Long noncoding RNA GATA2AS influences human erythropoiesis by transcription factor and chromatin landscape modulation. Blood *143*, 2300–2313. https://doi.org/10.1182/blood.2023021287.

Liu, M.Y., Lin, K.R., Chien, Y.L., Yang, B.Z., Tsui, L.Y., Chu, H.P.C., and Wu, C.S.P. (2024b). ATR phosphorylates DHX9 at serine 321 to suppress R-loop accumulation upon genotoxic stress. Nucleic Acids Res. *52*, 204–222. https://doi.org/10.1093/nar/gkad973.

Mendelson, A., and Frenette, P.S. (2014). Hematopoietic stem cell niche maintenance during homeostasis and regeneration. Nat. Med. *20*, 833–846. https://doi.org/10.1038/nm.3647.

Meng, Y., Carrelha, J., Drissen, R., Ren, X., Zhang, B., Gambardella, A., Valletta, S., Thongjuea, S., Jacobsen, S.E., and Nerlov, C. (2023). Epigenetic programming defines haematopoietic stem cell fate restriction. Nat. Cell Biol. *25*, 812–822. https://doi.org/10.1038/s41556-023-01137-5.

Meng, Y., and Nerlov, C. (2025). Epigenetic regulation of hematopoietic stem cell fate. Trends Cell Biol. *35*, 217–229. https://doi.org/10.1016/j.tcb.2024.08.005.

Morrison, S.J., and Scadden, D.T. (2014). The bone marrow niche for haematopoietic stem cells. Nature *505*, 327–334. https://doi.org/10.1038/nature12984.

Moshref, M., Questa, M., Lopez-Cervantes, V., Sears, T.K., Greathouse, R.L., Crawford, C.K., and Kol, A. (2021). Panobinostat Effectively Increases Histone Acetylation and Alters Chromatin Accessibility Landscape in Canine Embryonic Fibroblasts but Does Not Enhance Cellular Reprogramming. Front. Vet. Sci. *8*, 716570. https://doi.org/10.3389/fvets.2021.716570.

Mould, A.W., Morgan, M.A.J., Nelson, A.C., Bikoff, E.K., and Robertson, E.J. (2015). Blimp1/Prdm1 Functions in Opposition to Irf1 to Maintain Neonatal Tolerance during Postnatal Intestinal Maturation. PLoS Genet. *11*, e1005375. https://doi.org/10.1371/journal.pgen.1005375.

Nakajima, T., Uchida, C., Anderson, S.F., Lee, C.G., Hurwitz, J., Parvin, J.D., and Montminy, M. (1997). RNA helicase A mediates association of CBP with RNA polymerase II. Cell *90*, 1107–1112. https://doi.org/10.1016/s0092-8674(00)80376-1.

Närvä, E., Rahkonen, N., Emani, M.R., Lund, R., Pursiheimo, J.P., Nästi, J., Autio, R., Rasool, O., Denessiouk, K., Lähdesmäki, H., et al. (2012). RNA-Binding Protein L1TD1 Interacts with LIN28 via RNA and is Required for Human Embryonic Stem Cell Self-Renewal and Cancer Cell Proliferation. Stem Cell. *30*, 452–460. https://doi.org/10.1002/stem.1013.

Ren, X., Liu, Q., Zhou, P., Zhou, T., Wang, D., Mei, Q., Flavell, R.A., Liu, Z., Li, M., Pan, W., and Zhu, S. (2024). DHX9 maintains epithelial homeostasis by restraining R-loop-mediated genomic instability in intestinal stem cells. Nat. Commun. *15*, 3080. https://doi.org/10.1038/s41467-024-47235-2.

Ren, X., Wang, D., Zhang, G., Zhou, T., Wei, Z., Yang, Y., Zheng, Y., Lei, X., Tao, W., Wang, A., et al. (2023). Nucleic DHX9 cooperates with STAT1 to transcribe interferon-stimulated genes. Sci. Adv. *9*, eadd5005. https://doi.org/10.1126/sciadv.add5005.

Roch, A., Trachsel, V., and Lutolf, M.P. (2015). Brief Report: Single-Cell Analysis Reveals Cell Division-Independent Emergence of Megakaryocytes From Phenotypic Hematopoietic Stem Cells. Stem Cell. *33*, 3152–3157. https://doi.org/10.1002/stem.2106.

Singh, S.K., Singh, S., Gadomski, S., Sun, L., Pfannenstein, A., Magidson, V., Chen, X., Kozlov, S., Tessarollo, L., Klarmann, K.D., and Keller, J.R. (2018). Id1 Ablation Protects Hematopoietic Stem Cells from Stress-Induced Exhaustion and Aging. Cell Stem Cell *23*, 252–265.e8. https://doi.org/10.1016/j.stem.2018.06.001.

Suda, T., Takubo, K., and Semenza, G.L. (2011). Metabolic regulation of hematopoietic stem cells in the hypoxic niche. Cell Stem Cell *9*, 298–310. https://doi.org/10.1016/j.stem.2011.09.010.

Verovskaya, E.V., Dellorusso, P.V., and Passegué, E. (2019). Losing Sense of Self and Surroundings: Hematopoietic Stem Cell Aging and Leukemic Transformation. Trends Mol. Med. *25*, 494–515. https://doi.org/10.1016/j.molmed.2019.04.006.

Weinert, B.T., Narita, T., Satpathy, S., Srinivasan, B., Hansen, B.K., Schölz, C., Hamilton, W.B., Zucconi, B.E., Wang, W.W., Liu, W.R., et al. (2018). Time-Resolved Analysis Reveals Rapid Dynamics and Broad Scope of the CBP/p300 Acetylome. Cell *174*, 231–244.e12. https://doi.org/10.1016/j.cell.2018.04.033.

Wilkinson, A.C., Igarashi, K.J., and Nakauchi, H. (2020). Haematopoietic stem cell self-renewal in vivo and ex vivo. Nat. Rev. Genet. *21*, 541–554. https://doi.org/10.1038/s41576-020-0241-0.

Xiong, Y., Chen, Y., Luo, H., Shi, M., Wang, C., Hu, X., and Zhu, H. (2025). DHX9 as a prognostic biomarker and its biological roles in acute myeloid leukemia. BMC Cancer *25*, 1464. https://doi.org/10.1186/s12885-025-14708-6.

Stem Cell Reports, Volume *21*

Supplemental Information

DHX9 sustains hematopoietic stem cell function in cooperation with H3 acetylation

Minhui Shi, Mengqing Gao, Huixin Luo, Chong Wang, Xueyang Hu, Yacen Xiong, Yan Chen, Xingxing Ren, Shu Zhu, and Huaiping Zhu

Supplemental Information

DHX9 sustains hematopoietic stem cell function in cooperation with H3 acetylation

Minhui Shi, Mengqing Gao, Huixin Luo, Chong Wang, Xueyang Hu, Yacen Xiong, Yan Chen, Xingxing Ren, Shu Zhu, and Huaiping Zhu

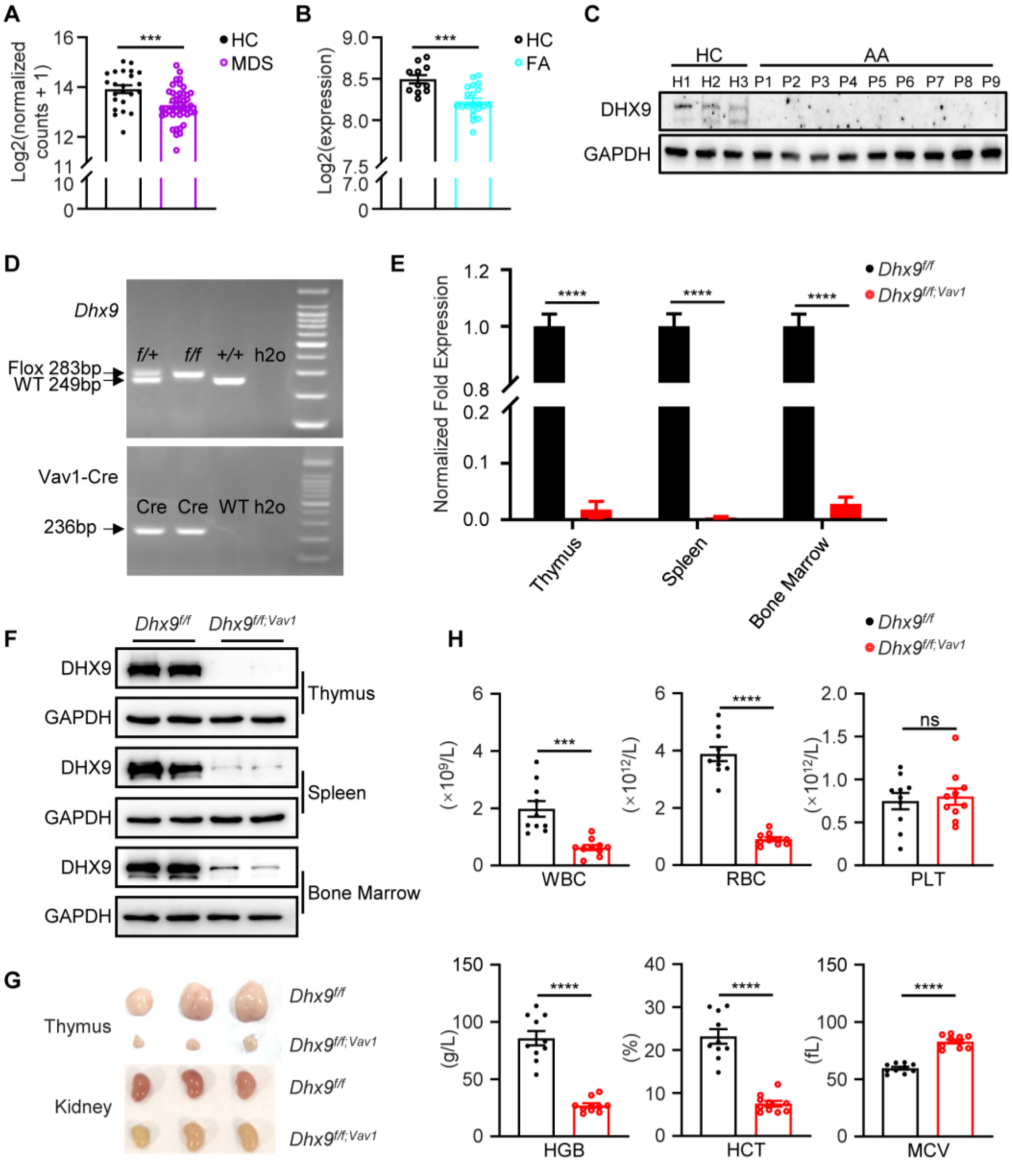

Figure S1. Generation of hematopoietic-specific *Dhx9* conditional knockout mice, related to Figure 1.

(A) RNA-seq data of $CD34^{+}$ cells from healthy controls (HC) (n = 23) and myelodysplastic syndromes (MDS) (n = 44) patients were obtained from the GEO database (GSE111085).

(B) mRNA expression profiles of BM cells from healthy controls and patients with fanconi anemia (FA) were obtained using the Affymetrix GPL96 microarray platform (GSE16334).

(C) Western blot showing DHX9 protein expression in BM samples from healthy controls (HC) and patients with aplastic anemia (AA).

(D) PCR Genotyping.

(E) qRT-PCR analysis to evaluate the deletion efficiency of *Dhx9* at the mRNA level. qRT-PCR primer pairs are designed and used for qRT-PCR analysis (n = 4).

(F) Western blot analysis of thymus, spleen and BM cells to confirm the DHX9 knockout efficiency at the protein level.

(G) Representative images showing smaller thymus and pale kidney from *Dhx9*$^{f/f;Vav1}$ mice (n = 3).

(H) Blood counts. White blood cell (WBC), red blood cell (RBC) and platelet (PLT) counts, hemoglobin (HGB), hematocrit (HCT) and mean corpuscular volume (MCV) were assessed within 2 weeks in control and *Dhx9* cKO mice (n = 10).

All mice used in the experiments were 7–14 days old. The data are presented as the mean ± SEM. ***P < 0.001; ****P < 0.0001; ns, not statistically significant; unpaired two-tailed Student's t-test.

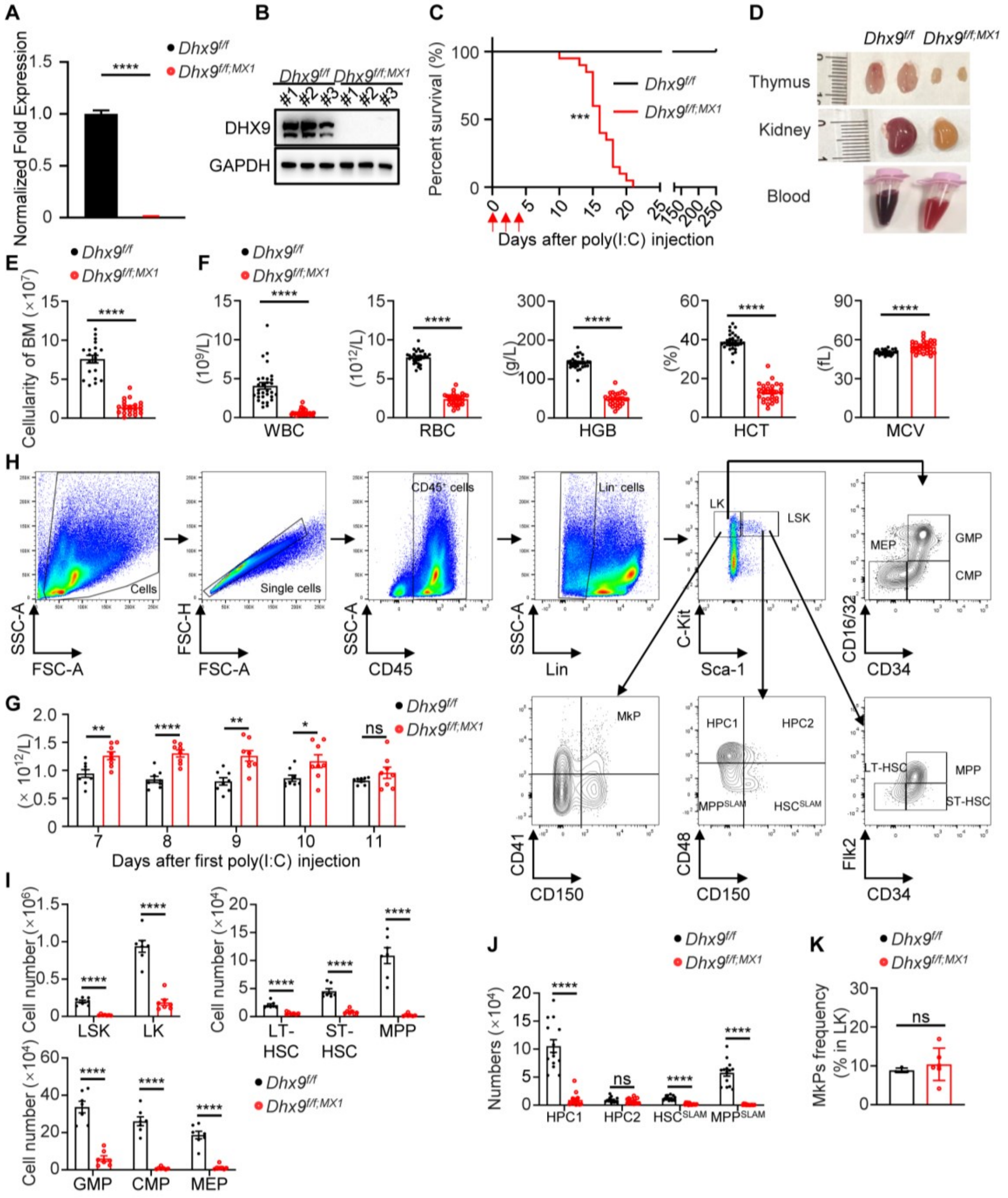

Figure S2. *Dhx9*$^{f/f;MX1}$ mice have the similar phenotypes to *Dhx9*$^{f/f;Vav1}$ mice, related to Figure 2.
(A) qRT-PCR analysis of *Dhx9* expression in BM cells from *Dhx9*$^{f/f}$ and *Dhx9*$^{f/f;MX1}$ mice (n = 4).
(B) Western blot analysis of DHX9 expression in BM cells from *Dhx9*$^{f/f}$ and *Dhx9*$^{f/f;MX1}$ mice (n = 3); GAPDH was used as a loading control.
(C) Kaplan–Meier survival curve of mice after poly(I:C)-induced *Dhx9* deletion, with red arrows marking the time points of poly(I:C) administration (n = 20).
(D) Representative images showing smaller thymus, pale kidney and blood from *Dhx9* cKO mice.
(E) Whole bone-marrow cellularity of *Dhx9*$^{f/f}$ (n = 20) and *Dhx9*$^{f/f;MX1}$ mice (n = 21).
(F) The routine blood parameters of *Dhx9*$^{f/f}$ (n = 30) and *Dhx9*$^{f/f;MX1}$ (n = 29) mice.
(G) Starting seven days after the first poly(I:C) injection, peripheral blood platelet counts were measured daily for five consecutive days (n = 8).
(H) Representative gating strategies used in FACS analyses for the frequencies of HSCs, LSK cells, and indicated lineage progenitors in mice.
(I and J) Absolute numbers of different hematopoietic stem and progenitor cell populations in the bone marrow from *Dhx9*$^{f/f}$ and *Dhx9*$^{f/f;MX1}$ mice (n ≥ 7).
(K) The proportion of $CD41^{+}CD150^{+}$ cells within the LK population was analyzed on day 11 following the first poly(I:C) injection in *Dhx9*$^{f/f}$ (n = 4) and *Dhx9*$^{f/f;MX1}$ mice (n = 6).
All mice used in these experiments except (K) were analyzed seven days after the first poly(I:C) injection. The data are presented as the mean ± SEM. **P* < 0.05; ***P* < 0.01; ****P* < 0.001; *****P* < 0.0001; ns, not statistically significant; statistical significance was determined using the log-rank test for (C) and unpaired two-tailed Student's *t*-test for (A), (E-G) and (I-K).

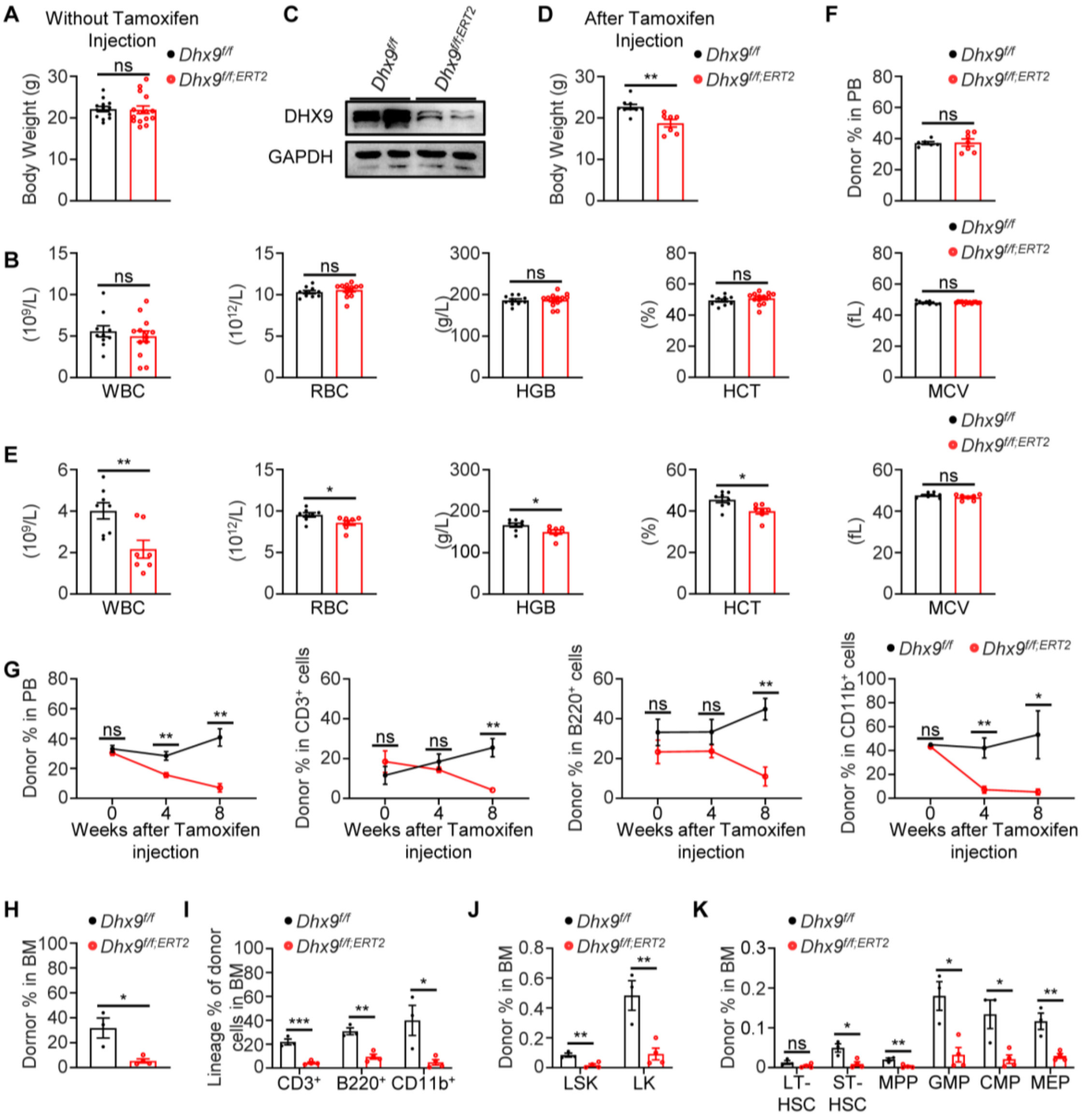

Figure S3. DHX9 sustains HSC for long-term hematopoiesis, related to Figure 3.
(A) Body weights of *Dhx9*$^{f/f}$ (n = 13) and *Dhx9*$^{f/f;ERT2}$ (n = 15) mice without tamoxifen injection.
(B) PB analysis of *Dhx9*$^{f/f}$ (n = 10) and *Dhx9*$^{f/f;ERT2}$ (n = 13) mice without tamoxifen injection.
(C) Western blot analysis of DHX9 expression in BM cells from *Dhx9*$^{f/f}$ and *Dhx9*$^{f/f;ERT2}$ mice after tamoxifen injection.
(D) Body weights of *Dhx9*$^{f/f}$ (n = 9) and *Dhx9*$^{f/f;ERT2}$ (n = 7) mice after tamoxifen injection.
(E) PB analysis of *Dhx9*$^{f/f}$ (n = 8) and *Dhx9*$^{f/f;ERT2}$ (n = 7) mice after tamoxifen injection.
(F) Donor chimerism in the PB of recipient mice was measured at four weeks after BMT without tamoxifen injection, and the results are graphed. *Dhx9*$^{f/f}$ (n = 6) and *Dhx9*$^{f/f;ERT2}$ (n = 7).
(G to K) The contributions of CD45.2$^+$ donor-derived cells in total CD45$^+$, T (CD3$^+$), B (B220$^+$) and myeloid (CD11b$^+$) cells from PB (G) or BM (H, I) and LSK, LK, HSC and other hematopoietic populations in the BM (J, K) were analyzed in primary recipients after tamoxifen injection. *Dhx9*$^{f/f}$ (n = 3) and *Dhx9*$^{f/f;ERT2}$ (n = 4).
The mice shown in (C, D and E) were used two weeks after the first tamoxifen injection. The data are presented as the mean ± SEM. *P < 0.05; **P < 0.01; ***P < 0.001; ns, not statistically significant; unpaired two-tailed Student's *t*-test.

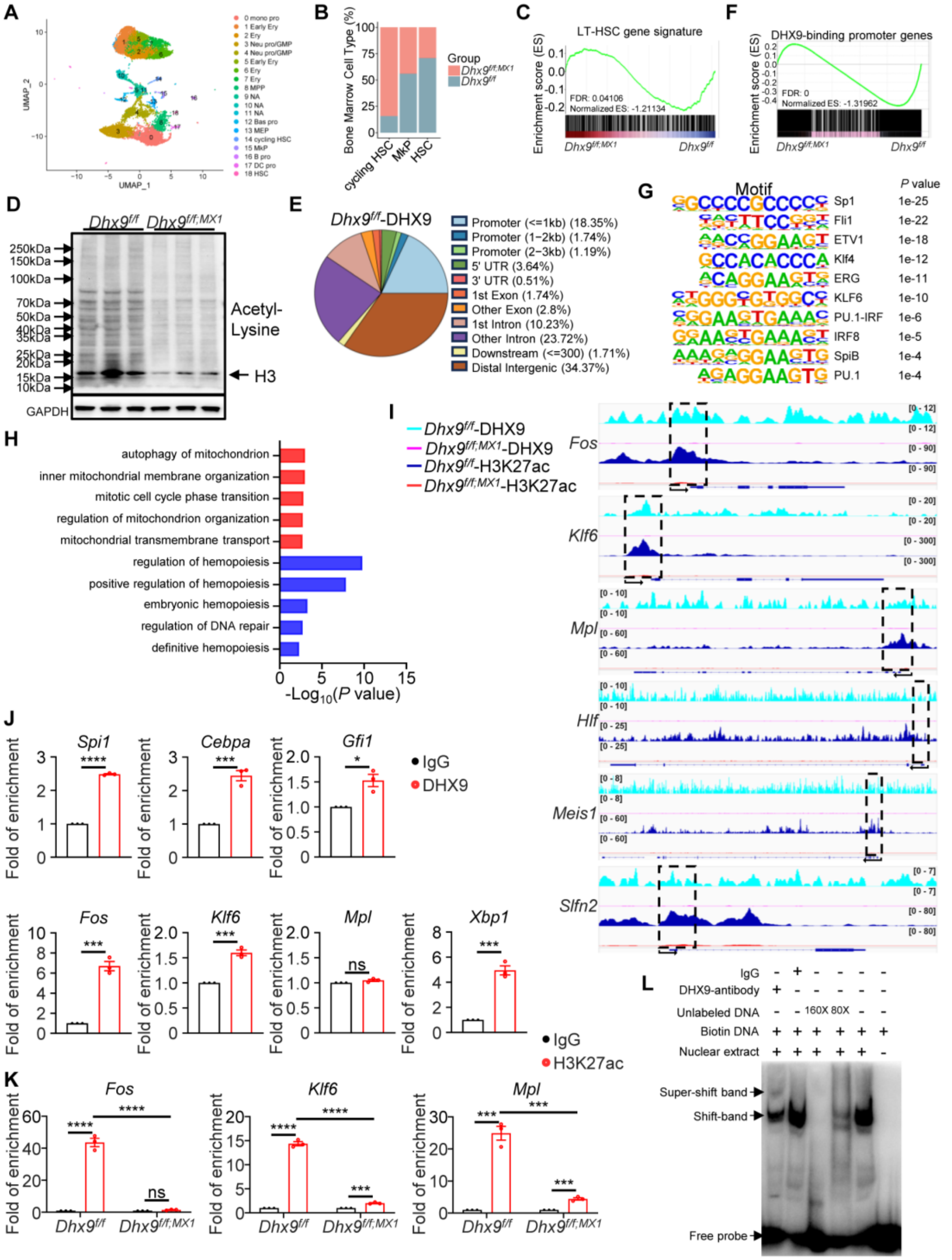

Figure S4. DHX9 promotes histone acetylation for transcriptional activation, related to Figure 5.
(A) UMAP representation of scRNA-seq data from combined Lin$^-$c-Kit$^+$ cells from BM of *Dhx9$^{f/f}$* and *Dhx9$^{f/f;MX1}$* mice.

(B) Proportion of *Dhx9*$^{f/f}$ and *Dhx9*$^{f/f;MX1}$ cells within each annotated cluster.
(C) GSEA was performed using genes ranked by $\log_2$ fold change between *Dhx9*$^{f/f;MX1}$ and *Dhx9*$^{f/f}$ HSC cluster cells. ES, enrichment score; FDR, false discovery rate.
(D) Western blot analyses of pan-lysine acetylation in TNCs from BM of Dhx9$^{f/f}$ and *Dhx9*$^{f/f;MX1}$ mice.
(E) Pie chart showing genomic annotation of DHX9-associated peaks around genes.
(F) Leading edge plot showing the enrichment of genes associated with DHX9-binding promoter genes in the RNA-seq data from *Dhx9*$^{f/f}$ and *Dhx9*$^{f/f;MX1}$ BM Lin$^-$ cells. ES, enrichment score; FDR, false discovery rate.
(G) Transcription factor motifs analysis enriched in DHX9-enriched promoter peak genes.
(H) Pathway analysis of genes associated with H3K27ac downregulated (blue) or upregulated (red) regions in *Dhx9*$^{f/f;MX1}$ Lin$^-$ cells.
(I) IGV genome browser tracks showing ChIP-seq data for DHX9 (*Dhx9*$^{f/f}$ Lin$^-$ cells)–, DHX9 (*Dhx9*$^{f/f;MX1}$ Lin$^-$ cells)–, H3K27ac (*Dhx9*$^{f/f}$ Lin$^-$ cells)–, and H3K27ac (*Dhx9*$^{f/f;MX1}$ Lin$^-$ cells)–bound promoter regions of hematopoiesis-related genes.
(J) ChIP-qPCR analysis of DHX9–bound sites in hematopoiesis-related gene promoters in *Dhx9*$^{f/f}$ Lin$^-$ cells (n = 3).
(K) ChIP-qPCR analysis of H3K27ac–bound sites in hematopoiesis-related gene promoters with or without *Dhx9* (n = 3).
(L) Super-shift electrophoretic mobility shift assay (EMSA) for the analysis of DHX9 binding on Xbp1 promoter region (n = 3), with data obtained from three independent experiments.
The data are presented as the mean ± SEM. **P* < 0.05; ****P* < 0.001; *****P* < 0.0001; ns, not statistically significant; unpaired two-tailed Student's *t*-test.

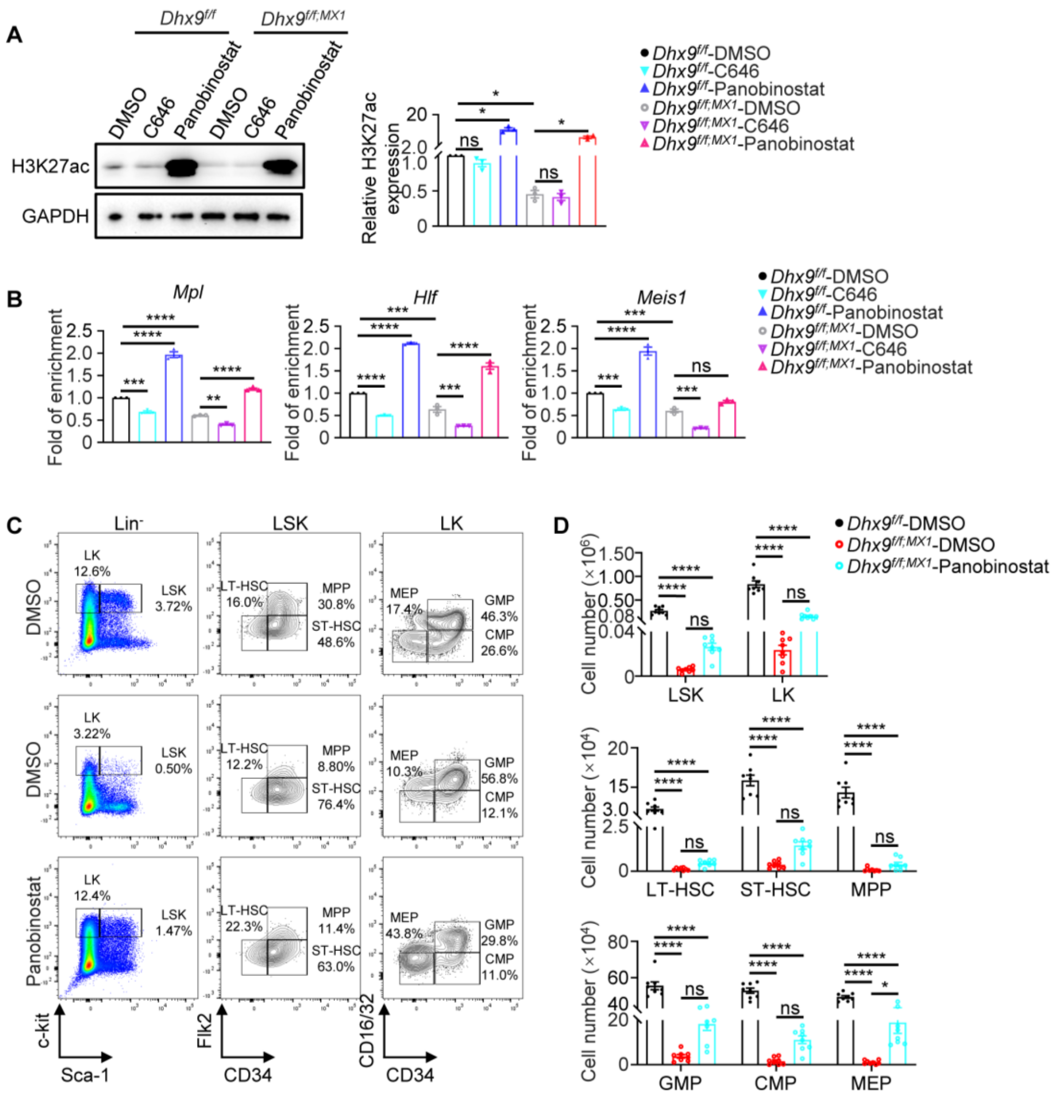

Figure S5. Increasing the level of H3K27ac partially rescue *Dhx9*-deficient HSCs in mice, related to Figure 5.

(A) BM cells isolated from *Dhx9*$^{f/f}$ or *Dhx9*$^{f/f;MX1}$ mice were treated with 40 μM C646 or 20 nM panobinostat for 48 h, then cells were lysed and subjected to immunoblotting analysis (left panel, n=3). The right panel shows the quantitative data of H3K27ac signal from three independent experiments.

(B) qRT-PCR analysis of hematopoiesis-related genes in *Dhx9*$^{f/f}$ or *Dhx9*$^{f/f;MX1}$ hematopoietic cells after treated with C646 or panobinostat (n = 3).

(C) Representative gating strategies used in FACS analyses for the frequencies of HSCs, LSK cells, and indicated lineage progenitors in mice following DMSO or 5 mg/kg panobinostat treatment.

(D) Absolute numbers of HSPC populations in the bone marrow of DMSO- or 5 mg/kg panobinostat-treated mice (n = 8).

The data are presented as the mean ± SEM. **P* < 0.05; ***P* < 0.01; ****P* < 0.001; *****P* < 0.0001; ns, not statistically significant; one-way ANOVA with Tukey's test (A, B and D).

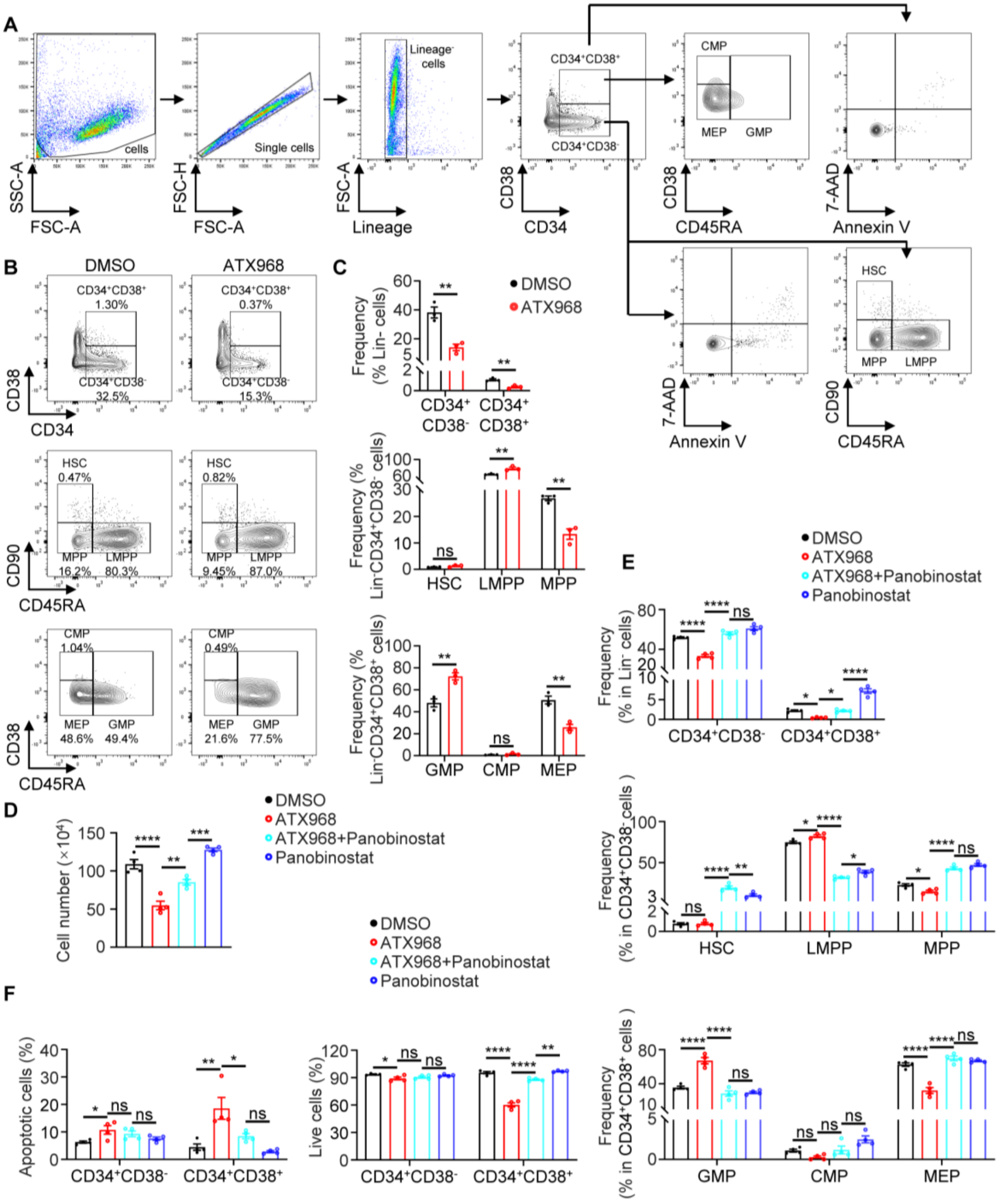

Figure S6. Inhibition of DHX9 in umbilical cord blood compromises HSC survival, related to Figure 6.

(A) Representative FACS gating strategies used to analyze the frequencies of HSCs and indicated lineage progenitors in human UCB cells.

(B and C) Representative FACS plots (B) and frequencies (C) of HSCs and indicated lineage progenitors in UCB CD34$^+$ cells following 6-day treatment with DMSO or ATX968 (n = 3).

(D) Total cell counts in UCB CD34$^+$ cells following 6-day treatment with DMSO, 40 μM ATX968, and/or 1 nM panobinostat (n = 4).

(E) Frequencies of HSCs and indicated lineage progenitors in UCB $CD34^+$ cells following 6-day treatment with DMSO, 40 μM ATX968, and/or 1 nM panobinostat (n = 4).
(F) Apoptotic cell ratios of $CD34^+CD38^-$ and $CD34^+CD38^+$ populations in UCB $CD34^+$ cells after 6-day treatment with DMSO, 40 μM ATX968, and/or 1 nM panobinostat (n = 4).
The data are presented as the mean ± SEM. **P* < 0.05; ***P* < 0.01; ****P* < 0.001; *****P* < 0.0001; ns, not statistically significant; unpaired two-tailed Student’s *t*-test (C), one-way ANOVA with Tukey’s test (D-F).

Table S1. List of qRT-PCR primers used in this study.

	Forward	Reverse
Gapdh	CTTCATTGACCTCAACTACATGG	CTCGCTCCTGGAAGATGGTGAT
Dhx9	GTTCTTTCGCCGTTCTCGTG	GGTGTCTTCTTCACACCCTCC
Spi1	GACCAGGAACGAGAGCCATC	TCTGCAGCTCTGTGAAGTGG
Cebpa	CAAGAACAGCAACGAGTACCG	GTCACTGGTCAACTCCAGCAC
Gfi1	CCCTTTGCGTGCGAGATGT	CAGCGTGGATGACCTCTTGAA
Fos	TTTCAACGCCGACTACGAGG	TCTGCGCAAAAGTCCTGTGT
Hlf	TTCCCGATGATTTGAAGCAGGA	CCGGATTGCGATCTGGTTCT
Meis1	AAGGTGATGGCTTGGACAAC	TGTGCCAACTGCTTTTTCTG
Xbp1	TCCGCAGCACTCAGACTATG	TGCCCAAAAGGATATCAGACTCA
Klf6	GAAAGCACATCAGCGCACTC	GACCTGGAGAAACACCTGTCA
Mpl	TTGTGGATAGTGTGGGCCTG	CCATAGCGGAGTTCATGCCT
Slfn2	AACCACAGAACTCAGAGGAATG	GCTCCGAGATTTAGACCCAGC

Table S2. List of ChIP-qPCR primers used in this study.

	Forward	Reverse
ChIP-*Spi1*	CTGCTTCGACTTCCCCCTTT	ACTCACCTCTCGTTCCTGGT
ChIP-*Cebpa*	GGTGGTGTCCCGAACACTTG	CAGGCCAGAGCGATAGGATT
ChIP-*Gfi1*	TCAGCTTCGTTGGGCGTATT	GTCCGCAGAAAGAAACCTGC
ChIP-*Fos*	AATCCTACACGCGGAAGGTC	GCGCTCTGTCGTCAACTCTA
ChIP-*Xbp1*	GCCTTCACGGATATCGCTCT	CCCGTTGCGTCATATGCTTC
ChIP-*Klf6*	CCGAGTTCGAATTGCACCAA	ATGTCAGCTCGCACCAATCG
ChIP-*Mpl*	ACACAGTGCCGGAGAAGATG	TGGTGACTTGTGCCTGGTTT

Table S3. Key resources table.

REAGENT OR RESOURCE	SOURCE	IDENTIFIER
Antibodies		
Rabbit monoclonal anti-GAPDH	Cell Signaling Technology	Cat# 2118; RRID:AB_561053
Rabbit polyclonal anti-DHX9	Abcam	Cat# ab26271; RRID:AB_777725
Rabbit polyclonal anti-DHX9	Abcam	Cat# ab70777; RRID:AB_1270326
Rabbit monoclonal anti-H3K9ac	Cell Signaling Technology	Cat# 9649; RRID:AB_823528
Rabbit polyclonal anti-H3K27ac	Abcam	Cat# ab4729; RRID:AB_2118291
Rabbit monoclonal anti-IgG	Cell Signaling Technology	Cat# 3900; RRID:AB_1550038
Rabbit monoclonal anti-HA	Cell Signaling Technology	Cat# 3724; RRID:AB_1549585
Rabbit monoclonal anti-CBP	Cell Signaling Technology	Cat# 7389; RRID:AB_2616020
Mouse monoclonal anti-p300	Santa Cruz Biotechnology	Cat# sc-32244; RRID:AB_628076
Alexa Fluor™ Plus 647 anti-rabbit IgG	Thermo Fisher Scientific	Cat# A32733; RRID:AB_2633282
Alexa Fluor™ 488 anti-mouse IgG	Thermo Fisher Scientific	Cat# A11029; RRID:AB_2534088
DAPI Solution	BD Biosciences	Cat# 564907; RRID:AB_2869624
FITC anti-mouse Lineage Cocktail	BioLegend	Cat# 133301; RRID: AB_10697030
Brilliant Violet 510™ anti-mouse CD45	BioLegend	Cat# 103138; RRID:AB_312973
PE/Cyanine7 anti-mouse CD45.1	BioLegend	Cat# 110730; RRID:AB_1134168
Alexa Fluor® 700 anti-mouse CD45.2	BioLegend	Cat# 109822; RRID:AB_493731
APC anti-mouse c-kit	BioLegend	Cat# 105812; RRID:AB_313221
PE/Cyanine7 anti-mouse c-kit	BD Biosciences	Cat# 558163; RRID:AB_647250
Pacific Blue™ anti-mouse Sca-1	BioLegend	Cat# 122520; RRID:AB_2143237
Alexa Fluor 700 anti-mouse CD34	eBioscience	Cat# 56-0341-82; RRID: AB_493998
PE-CF594 anti-Mouse Flk2	BD Biosciences	Cat# 562537; RRID: AB_2737639
PE/Cy7 anti-mouse CD150	BioLegend	Cat# 115914; RRID:AB_439797
FITC anti-mouse CD48	BioLegend	Cat# 103404; RRID:AB_313019
APC anti-mouse CD48	BioLegend	Cat# 103411; RRID: AB_571996
FITC anti-mouse CD16/32	BioLegend	Cat# 101306; RRID:AB_312805
PE anti-mouse B220	BioLegend	Cat# 103208; RRID:AB_312993
PE anti-mouse CD3ε	BioLegend	Cat# 100308; RRID:AB_312673
PE anti-mouse CD2	BioLegend	Cat# 100108; RRID:AB_2073690
PE anti-mouse Gr-1	BioLegend	Cat# 108408; RRID:AB_313373
PE anti-mouse Ter-119	BioLegend	Cat# 116208; RRID:AB_313709
PE anti-Mouse CD41	BD Biosciences	Cat# 558040; RRID:AB_397004
eFluor 450 anti-mouse CD3	eBioscience	Cat# 48-0032-82; RRID: AB_1272193
FITC anti-mouse/human CD11b	BioLegend	Cat# 101206; RRID:AB_312789
Alexa Fluor® 488 anti-mouse/human Ki-67	BioLegend	Cat# 151204; RRID:AB_2566800
Alexa Fluor® 488 anti-H2A.X Phospho (Ser139)	BioLegend	Cat# 613405; RRID:AB_528914
Brilliant Violet 510™ anti-human Lineage Cocktail	BioLegend	Cat# 348807; RRID:AB_3097689
BV421 anti-human CD34	BD Biosciences	Cat# 562577; RRID:AB_2687922

Brilliant Violet 605™ anti-human CD90 (Thy1)	BioLegend	Cat# 328128; RRID:AB_2562281
APC anti-human CD38	eBioscience	Cat# 17-0389-42; RRID:AB_1834353
APC-eFluor™ 780 anti-human CD45RA	eBioscience	Cat# 47-0458-42; RRID:AB_10853641
Chemicals, peptides, and recombinant proteins		
ACK lysis buffer	Gibco	Cat# A1049201
TRIzol	Invitrogen	Cat# 15596018CN
Protease inhibitor cocktail	Sigma-Aldrich	Cat# 11873580001
DMSO	Sigma	Cat# D2650
poly (I:C)	Sigma	Cat# P1530
Tamoxifen	Sigma	Cat# T5648
C646	Sigma	Cat# 382113
Panobinostat	Selleckchem	Cat# LBH589
ATX968	TargetMol	Cat# T82568
Protein G Sepharose 4 Fast Flow	GE Health	Cat# 17-0618-01
StemSpan™ SFEM II	StemCell Technologies	Cat# 09655
Methocult GF M3434	StemCell Technologies	Cat# 03434
MethoCult™ H4435	StemCell Technologies	Cat# 04435
Murine SCF	Peprotech	Cat# 250-03
Murine IL3	Peprotech	Cat# 213-13
Murine FLT3L	Peprotech	Cat# 250-31L
Murine IL6	Peprotech	Cat# 216-16
Human SCF	Peprotech	Cat# 300-07-10UG
Human FLT3L	Peprotech	Cat# 300-19-10UG
Human TPO	Peprotech	Cat# 300-18-10UG
Human IL3	Peprotech	Cat# 200-03-10UG
Human IL6	Peprotech	Cat# 200-06-20UG
Fetal Bovine Serum	Bioind	Cat# 04-001-1A
Polyethylenimine	Polysciences	Cat# 23966
Critical commercial assays		
Human CD34 MicroBead Kit	Miltenyi Biotec	Cat# 130-046-702
Lineage Cell Depletion Kit mouse	Miltenyi Biotec	Cat# 130-090-858
CD117 MicroBeads, Miltenyi Biotec	Miltenyi Biotec	Cat# 130-091-224
FITC Annexin V Apoptosis Detection Kit with 7-AAD	BioLegend	Cat# 640922
Foxp3/Transcription Factor Staining Buffer Set	eBioscience	Cat# 00-5523
Taq Pro Universal SYBR qPCR Master Mix	Vazyme	Cat# Q712-03
PrimeScript™ RT Master Mix	Takara	Cat# RR036A
CellROX® Oxidative Stress Reagents	Invitrogen	Cat# C10444
Hyperactive Universal CUT&Tag Assay Kit for Illumina Pro	Vazyme	Cat# TD904-01
Immobilon Western Chemiluminescent HRP Substrate	Millipore	Cat# WBKLS0500
Pierce™ BCA Protein Assay Kits	Thermo Fisher	Cat# 23225

Supplemental methods

293T transient transfection

293T cells (HyCyte, TCH-C101) were cultured in DMEM supplemented with 10% FBS and 1% penicillin-streptomycin. The plasmids pLVX-*DHX9*-IRES-ZsGreen and pEnCMV-*CREBBP*-HA-SV40-Neo (MiaoLing Biology, P29740) were co-transfected using polyethylenimine.

Cell apoptosis, cell cycle and DNA damage staining

Briefly, for cell apoptosis analysis, stained HSPCs were stained for 15 min with the FITC-labeled Annexin V and 7-AAD Viability Staining Solution in Annexin V binding buffer. For nuclear protein staining, stained HSPCs were further fixed and permeabilized using a Cytofix/Cytoperm kit. For cell cycle analysis, cells were stained with an anti-Ki67 antibody and DAPI. For DNA damage analysis, cells were stained with anti-γH2A.X antibody.

Determination of reactive oxygen species (ROS) generation

Briefly, BM cells from WT or $Dhx9^{f/f;MX1}$ mice were harvested and stained with HSPCs markers, including lineage cocktail, antibodies against CD45, Sca-1, CD34, Flk2, c-Kit. For analysis of ROS, cells were stained with 5 μM CellRox in 1× Phosphate buffer saline (PBS) at 37°C for 30 minutes, and then resuspended in warm PBS to assess the ROS intermediate levels.

Colony-forming unit assay

Freshly isolated mouse $Lin^-c\text{-}Kit^+$ cells (2×10^3 cells) and human UCB $CD34^+$ cells (4×10^4 cells) were suspended in 100 μL IMDM supplemented with 2% FBS and plated in 1 mL of methylcellulose-based medium. Mouse BM cells were cultured in MethoCult™ GF M3434, while UCB $CD34^+$ cells were cultured in MethoCult™ GF H4435 with either DMSO or ATX968 (40 μM). All samples were cultured in triplicate and incubated at 37°C with 5% CO_2 and ≥ 95% humidity for 12 days. Hematopoietic colonies were counted under an inverted microscope. For both mouse and UCB samples, the mean of triplicates was used for final statistical analyses.

Confocal imaging

Lin⁻ BM cells were placed on glass slides. The cells were fixed with 4% paraformaldehyde for 15 min at RT, washed with PBS and then permeabilized with 0.3% Triton X-100 for 10 min. After being blocked with 5% BSA for 1 h at RT, cells were stained overnight at 4°C with primary antibodies, followed by incubation with secondary antibodies for 1 h at RT. The primary antibodies were anti-DHX9, anti-p300, anti-H3K27ac. The secondary antibodies were goat anti-mouse Alexa Fluor™ 488 and/or goat anti-rabbit Alexa Fluor™ Plus 647. After washing, cells mounted with DAPI. Fluorescence was examined using a confocal microscope (Zeiss, LSM800) and staining intensity was quantified by ImageJ program.

Primary cell culture and chemical treatment

In mice, total BM cells were harvested and cultured in HSC expansion media consisting of StemSpan SFEM supplemented with murine 50 ng/ml SCF, 20 ng/ml Flt-3 ligand, 10 ng/ml IL-3, and 10 ng/ml IL-6 after a quick RBC lysis. C646 and the HDACi panobinostat were used in BM cells at 40 μM and 20 nM respectively.

All umbilical cord blood units used were obtained under the approval of the Ethics Committee of the First Affiliated Hospital of USTC (2023-RE-388). Human UCB $CD34^+$ cells were isolated using the human CD34 MicroBead Kit. These cells were cultured in HSC expansion media consisting of StemSpan SFEM supplemented with human 100 ng/ml SCF, 100 ng/ml Flt-3 ligand, 50 ng/ml thrombopoietin, 10 ng/ml IL-3, and 10 ng/ml IL-6. DHX9 specific inhibitor ATX968 and HDACi panobinostat were used in $CD34^+$ cord blood cells at 40 μM and 1 nM respectively.

Protein extraction and immunoblot analysis

Human BM samples were obtained under the approval of the Ethics Committee of the First Affiliated Hospital of USTC (2023-RE-307). Cells were harvested by centrifugation at 500 × g for 5 min, washed with cold PBS and lysed in NP-40 lysis buffer supplemented with 1% protease inhibitor cocktail on ice for 30 min, then centrifuged at 12000 × g for 15 min at 4°C to harvest the supernatant. Protein concentration was measured using a Nanophotometer (IMPLEN, NP80) with a BCA protein assay kit. Furthermore, equal amounts of protein extracts were fractionated on 4%~12% gradient SDS–PAGE gels and transferred to PVDF membranes. The blots were blocked with 5% nonfat milk at RT for 1 hour and then incubated with

primary antibodies overnight at 4°C. After incubation, membranes were washed with TBST, and then incubated with secondary antibody for 1 h at RT. Bands were captured using the FluorChem M system (ProteinSimple, USA).

For immunoprecipitation, 293T cells were collected at 48 h after transfection using lysis buffer [containing 50 mM Tris-HCl, (pH 7.4), 150 mM NaCl, 2 mM EDTA, 5% glycerol, and 0.5% NP-40] supplemented with PMSF and a protease inhibitor cocktail. The lysates were diluted to approximately 1 mg/ml with lysis buffer, 500 µg of the total cell lysate was incubated with the indicated antibody overnight at 4°C, protein A/G-Sepharose beads were added, and the mixture was further incubated for 1.5 h at 4°C. After centrifugation at 12000 × g for 30 s, the beads were washed 6 times with cell lysis buffer before analysis. The primary antibodies used in this study including anti-GAPDH, anti-DHX9, anti-H3K27ac, anti-H3K9ac, anti-HA, anti-CBP and anti-IgG.

RNA isolation and quantitative reverse transcription PCR (qRT-PCR) analysis

Total RNA was isolated using TRIzol reagent. The cDNA was synthesized using the PrimeScript™ RT Master Mix and subjected to qPCR with SYBR Green qPCR Master Mix. All primers used are listed in Table S1. *Gapdh* was used as an internal normalization control.

CUT&Tag assay and ChIP-qPCR analysis

CUT&Tag assays were performed according to the manufacturer's instructions. Briefly, prewashed Lin$^-$ cells were incubated with concanavalin A–coated magnetic beads, incubated with anti-DHX9, anti-H3K27ac, or normal anti-IgG primary antibodies for overnight at 4°C and incubated secondary antibodies at room temperature (RT) for 1 h. After three washes, the samples were incubated with pA/G-Tnp Pro at RT for 1 h. The extracted DNA fragments were subjected to PCR amplification, and then performed high-throughput sequencing. Clean reads were aligned to the mouse genome (GRCm39) via software Bowtie2 (version 2.2.7), and peak calling was done by using MACS3 (version 3.0.0a6). Different peaks were analyzed using edgeR (version 4.0.1) with a false discovery rate (FDR) < 0.05 and an absolute fold change ≥ 2. All libraries were sequenced on the Illumina Novaseq 6000 platform. ChIP-qPCR primers used in this study are listed in Table S2.

Super-shift electrophoretic mobility shift assay (EMSA)

Nuclear proteins (20 μg) were preincubated in the binding buffer with or without molar excess of unlabeled competitor double-stranded oligonucleotides or anti-DHX9 antibody at room temperature for 10 min. The 5′ biotin-TEG–labeled double-stranded DNA (dsDNA) probe corresponding to the XBP1 promoter region (5’ - GAACAGCTGTGCAGCCACGCTGGACA - 3’) was added, and the incubation was continued for 30 min. DNA–protein complexes were separated from free probes on a 6% native polyacrylamide gel at low temperature and detected by fluorescence imaging to assess binding specificity.

Single-cell (sc) RNA-seq, data preprocessing, and dimensionality reduction and data analysis

Freshly isolated mouse $Lin^{-}c\text{-}Kit^{+}$ cells with >90% viability were used to generate scRNA-seq libraries using the 10x Genomics Chromium Single Cell 3′ v3 kit, followed by sequencing on an Illumina NovaSeq 6000 platform. The raw sequencing reads were de-multiplexed and aligned to the mouse genome (GRCm39) using Cell Ranger (v6.0.2). The resulting gene-cell expression matrices were imported into R (v4.5.1) for downstream analysis using the *Seurat* (v4.3.0) and *SeuratObject* (v4.1.3) packages. Cells expressing fewer than three genes, showing high mitochondrial gene content, or identified as erythrocytes or doublets were removed. Gene expression matrices were normalized and scaled, and highly variable genes were identified for subsequent analyses. Principal component analysis (PCA) was then performed to capture major sources of biological variation, followed by clustering and visualization using the Uniform Manifold Approximation and Projection (UMAP) algorithm. Unsupervised cell clusters were annotated using the *scCATCH* package (v3.3.2), based on reference gene signatures and manually curated marker genes from previous studies (Dahlin et al., 2018; Kucinski et al., 2024; Poscablo et al., 2024; Rodriguez-Fraticelli et al., 2020).

Supplemental references

Dahlin, J.S., Hamey, F.K., Pijuan-Sala, B., Shepherd, M., Lau, W.W.Y., Nestorowa, S., Weinreb, C., Wolock, S., Hannah, R., Diamanti, E., et al. (2018). A single-cell hematopoietic landscape resolves 8 lineage trajectories and defects in Kit mutant mice. Blood 131, e1-e11. 10.1182/blood-2017-12-821413.

Kucinski, I., Campos, J., Barile, M., Severi, F., Bohin, N., Moreira, P.N., Allen, L., Lawson, H., Haltalli, M.L.R., Kinston, S.J., et al. (2024). A time- and single-cell-resolved model of murine bone marrow hematopoiesis. Cell Stem Cell 31, 244-259.e210. 10.1016/j.stem.2023.12.001.

Poscablo, D.M., Worthington, A.K., Smith-Berdan, S., Rommel, M.G.E., Manso, B.A., Adili, R., Mok, L., Reggiardo, R.E., Cool, T., Mogharrab, R., et al. (2024). An age-progressive platelet differentiation path from hematopoietic stem cells causes exacerbated thrombosis. Cell 187, 3090-3107.e3021. 10.1016/j.cell.2024.04.018.
Rodriguez-Fraticelli, A.E., Weinreb, C., Wang, S.W., Migueles, R.P., Jankovic, M., Usart, M., Klein, A.M., Lowell, S., and Camargo, F.D. (2020). Single-cell lineage tracing unveils a role for TCF15 in haematopoiesis. Nature 583, 585-589. 10.1038/s41586-020-2503-6.
